# Morphological and Hyperphosphorylation Transitions of Nanoscale Tau Aggregates in Alzheimer's Disease

**DOI:** 10.1002/advs.202509773

**Published:** 2025-11-19

**Authors:** Adriana N. Santiago‐Ruiz, Siewert Hugelier, Gabriela L. Correa, Charles R. Bond, Edward B. Lee, Melike Lakadamyali

**Affiliations:** ^1^ Biochemistry, Biophysics, and Chemical Biology Graduate Group University of Pennsylvania Philadelphia USA; ^2^ Department of Physiology University of Pennsylvania Philadelphia USA; ^3^ Department of Pathology and Laboratory Medicine University of Pennsylvania Philadelphia USA

**Keywords:** Alzheimer's disease, DNA‐PAINT, hyperphosphorylation, nano‐aggregates, super‐resolution microscopy, tau aggregation

## Abstract

Tau aggregation plays a critical role in Alzheimer's Disease (AD), where neurofibrillary tangles (NFTs) are a pathological hallmark. While much attention is given to NFTs, emerging evidence highlights nano‐sized tau oligomers as toxic entities. Using super‐resolution microscopy, we visualized nano‐sized tau aggregates (nano‐aggregates) in human postmortem brain tissues from intermediate and advanced AD, Primary Age‐Related Tauopathy (PART), and controls lacking tau pathology. Surprisingly, tau nano‐aggregates hyperphosphorylated at threonine 231 (p‐T231) and threonine 181 (p‐T181)are detected in control cases, whereas hyperphosphorylated serine 202/threonine 205 (p‐S202/T205)nano‐aggregates are specifically associated with AD and, to a lesser extent, observed in PART. This finding suggests that distinct hyperphosphorylation signatures distinguish physiological from pathological nano‐aggregates. Moreover, nano‐aggregates exhibit morphological differences between AD and non‐AD conditions, increasing in size and complexity in AD. In advanced AD, nano‐aggregates typically contain multiple distinct hyperphosphorylated residues, whereas intermediate AD nano‐aggregates are predominantly marked by a single hyperphosphorylated residue. These findings reveal novel transitions in the morphology and hyperphosphorylation states of tau nano‐aggregates as they shift from physiological to pathological forms. The ability to detect and profile physiological and pathological nanoscale tau aggregates in human brain tissues opens new avenues for studying the molecular underpinnings of tauopathies.

## Graphical abstract

Super‐resolution microscopy identifies nano‐sized tau aggregates (nano‐aggregates) and reveals distinct transitions in their morphology and phosphorylation profiles across control, Primary Age‐Related Tauopathy, and Alzheimer's disease brains. Morphology and hyperphosphorylation signatures distinguish physiological from pathological forms, with nano‐aggregates growing in size and complexity as disease advances. These findings provide new avenues for studying protein aggregates in tauopathies.

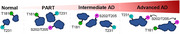

## 1 Introduction

Tau is a microtubule‐associated protein that plays a crucial role in microtubule stability and assembly^[^ ^1^, ^2^, ^3^ ^]^ as well as in regulating intracellular transport by motor proteins.^[^ ^4^, ^5^, ^6^, ^7^ ^]^ In a group of neurodegenerative diseases known as tauopathies, including Alzheimer's Disease (AD), tau becomes mislocalized and forms large, insoluble fibrillar inclusions within the somatodendritic compartment,^[^ ^1^, ^2^, ^3^, ^8^, ^9^, ^10^, ^11^ ^]^ such as the neurofibrillary tangles (NFTs) characteristic of AD.^[^ ^2^, ^12^, ^13^ ^]^ NFTs spread across the brain in a stereotypic pattern, beginning in the transentorhinal region in the medial temporal lobe (Braak stage 1), followed by the subiculum and hippocampal pyramidal cell layer (Braak stage 2), the entorhinal cortex with some extension into the neocortex (Braak stage 3), and the superior temporal cortex and frontal cortex (Braak stage 5).^[^ ^13^, ^14^, ^15^, ^16^, ^17^ ^]^ In later AD stages, NFT pathology intensifies in the hippocampus, extends to the neocortex, and eventually reaches the primary cortical areas.^[^ ^13^, ^14^, ^15^, ^16^ ^]^ Braak stages 4‐6 are strongly associated with clinically observable dementia, whereas Braak stages 1‐2 typically occur in individuals who are clinically asymptomatic.^[^ ^13^, ^14^, ^15^, ^16^, ^17^ ^]^ This progression, and its correlation with clinical decline, suggests that tau aggregation plays a key role in driving the pathogenesis of AD.^[^ ^2^, ^12^, ^18^ ^]^

Tau undergoes various post‐translational modifications (PTMs), which regulate both its normal physiological functions and its propensity to aggregate in disease.^[^ ^10^, ^19^, ^20^ ^]^ Among these PTMs, phosphorylation is particularly significant as NFTs isolated from human AD brain tissues consist of tau that is hyperphosphorylated, often referred to as phospho‐tau or p‐tau.^[^ ^21^, ^22^, ^23^ ^]^ The Proline‐Rich Region (PRR) of tau, located just before the Microtubule‐Binding Region (MTBR) and near the N‐terminus, is particularly susceptible to hyperphosphorylation.^[^ ^24^ ^]^ Given the critical role of hyperphosphorylation in disease, several antibodies targeting specific phospho‐epitopes have been developed.^[^ ^25^ ^]^ One of the most widely used is AT8, which detects tau phosphorylated at serine 202 and threonine 205 (p‐S202/T205).^[^ ^26^ ^]^ AT8 staining is the highest at the most advanced neuropathological stages of AD (Braak stages 5‐6).^[^ ^27^ ^]^ Hence, p‐S202/T205 is sometimes considered to be a marker of late‐stage tau aggregation. In contrast, hyperphosphorylation at threonine 231 (p‐T231), which is recognized by the AT180 antibody,^[^ ^28^, ^29^ ^]^ is associated with pre‐NFTs^[^ ^30^ ^]^ and is significantly increased in certain brain regions at earlier Braak stages (Braak stages 3‐4).^[^ ^27^ ^]^ As a result, p‐T231 is sometimes considered a marker of early‐stage tau aggregation. Another phosphorylation site of interest is threonine 181 (p‐T181). Increased p‐T181 levels have been observed during preclinical AD, when amyloid‐β pathology is still minimal,^[^ ^31^, ^32^ ^]^ suggesting its potential utility as an early biomarker of disease onset.

NFTs and other micron‐sized aggregates that are easily detectable using either silver stain or p‐tau antibodies and light microscopy were long considered as the toxic tau aggregates. However, in vitro and cell‐based studies have demonstrated that much smaller, nano‐sized tau aggregates, particularly soluble tau oligomers, impair memory, disrupt synaptic function, and cause neurodegeneration.^[^ ^33^, ^34^, ^35^, ^36^, ^37^, ^38^ ^]^ Notably, targeting tau oligomers has been shown to prevent cognitive impairment and tau toxicity.^[^ ^39^ ^]^ Consequently, current models of disease pathogenesis increasingly point to pre‐NFT aggregates, especially tau oligomers, as the primary toxic species that initiate tau aggregation and contribute to neurodegeneration.^[^ ^40^, ^41^, ^42^ ^]^ However, detecting these oligomers in intact brain tissue remains a major challenge, as their size falls below the diffraction limit of conventional light microscopy.

Super‐resolution microscopy overcomes the diffraction limit of conventional light microscopy, enabling the visualization and characterization of biological structures at the nanoscale.^[^ ^43^ ^]^ Using this approach, we recently examined tau aggregation in an engineered cell model that recapitulates key features of tauopathy‐associated tau aggregation.^[^ ^44^, ^45^ ^]^ Super‐resolution microscopy revealed the presence of tau oligomers in addition to insoluble polymorphous aggregates such as fibrils and NFT‐like aggregates.^[^ ^44^ ^]^ These findings demonstrate that super‐resolution light microscopy can successfully detect nanosized tau aggregates, such as tau oligomers and small fibrils, in engineered cell models. However, whether these nanometric tau aggregates can also be visualized and evaluated in human brain tissue remains an open question.

Here, we leveraged DNA‐PAINT super‐resolution imaging^[^ ^46^ ^]^ to visualize and quantify three disease‐relevant p‐tau epitopes, p‐S202/T205, p‐T181, and p‐T231 in nano‐sized (tau oligomers and small fibrils) and micron‐sized (large fibrils and NFTs) tau aggregates within human postmortem brain tissues from the temporal lobe of AD patients at different Braak stages (Braak 4 and 6). We compared these findings to tau‐pathology free controls and cognitively normal cases neuropathologically diagnosed with Primary Age‐Related Tauopathy (PART‐Braak stages 1, 2, 3), a common condition in aging brains characterized by the presence of tau pathology without amyloid‐beta pathology.^[^ ^47^ ^]^ Our findings reveal that nano‐sized tau aggregates, which we refer to as nano‐aggregates, are the most abundant p‐tau species across Braak stages, accounting for over 50% of all tau aggregates. These nano‐aggregates are consistent in size and abundance with tau oligomers detected by the oligomer‐specific antibody T22^[^ ^36^ ^]^ and likely represent a heterogeneous population of soluble oligomers and insoluble protofibrils. Surprisingly, p‐T181 and p‐T231 positive nano‐aggregates were detected in control cases, whereas p‐S202/T205 positive nano‐aggregates were specifically found in AD cases and, to a lesser extent, PART cases. These results demonstrate the high sensitivity of DNA‐PAINT in detecting both physiological and pathological p‐tau nano‐aggregates, even in regions without overt histological tau pathology and before the onset of clinical symptoms. Furthermore, we observed progressive changes in the morphology and multi‐site phosphorylation patterns of tau nano‐aggregates, as they transition from physiological to pathological forms. Our approach offers a powerful tool for characterizing the PTM profiles of tau aggregates across a range of tauopathies.

## 2 Results

### 2.1 Histological Characterization of Brain Tissue Samples

To visualize tau aggregates, we utilized formalin‐fixed, paraffin embedded human postmortem brain tissue sections provided by the University of Pennsylvania (Penn) Center for Neurodegenerative Disease Research (CNDR). Specifically, we used 6 µm thick sections from the temporal cortex of ten cases corresponding to PART (Braak stages 1‐3, N = 4 cases), intermediate AD (Braak stage 4, N = 3 cases), and advanced AD (Braak stage 6, N = 3 cases) (**Table** **1**; Figure S1A, Supporting Information). As controls, we obtained tissue sections from the frontal temporal region of two cases (38‐yo and 60‐yo) lacking any tau pathology (**Table** 1; Figure S1A, Supporting Information).

**Table 1:** Demographic, clinical, and pathological characteristics of studied case.

Case number	Sex	Age	Brain region	Clinical diagnosis	Pathological diagnosis	ThaI phase score	Braak stage	CERAD score	Post‐mortem interval
A	Female	38	Frontal Temporal	Normal	Unremarkable	0	0	0	3.5
B	Female	60	Frontal Temporal	Normal	Unremarkable	0	0	0	9
A	Female	56	Temporal Cortex	Normal	PART	0	1	0	12
A	Male	74	Temporal Cortex	PD	LBD & PART	0	2	0	30.5
B	Female	67	Temporal Cortex	Normal	PART	0	2	0	21
A	Female	87	Temporal Cortex	Schizophrenia	Schizophrenia & PART	0	3	0	33.5
A	Male	90+	Temporal Cortex	Normal	Intermediate Level of AD	2	4	1	18
B	Female	80	Temporal Cortex	Probable AD	Intermediate Level of AD & LATE	3	4	2	18
C	Female	90+	Temporal Cortex	Probable AD	Probable AD & LATE	3	4	2	18
A	Male	68	Temporal Cortex	Probable AD	High Level of AD	3	6	3	20
B	Male	66	Temporal Cortex	DLB	High Level of AD	3	6	3	73
C	Female	69	Temporal Cortex	FTLD‐PPA (Lopogenic)	High Level of AD	3	6	3	24

((Postmortem interval corresponds to the time in hours between death and specimen fixation))((Age equals age at death. Patients older than 90 years of age are listed as 90+ to protect their health information)); ((PART: Primary Age Related Tauopathy)); ((PD: Parkinson's Disease)); ((LBD: Brainstem‐Predominant Lewy Body Disease)); ((AD: Alzheimer's Disease)); ((LATE: Limbic‐predominant age‐related TDP‐43 encephalopathy)); ((DLB: Dementia with Lewy Bodies)); ((FTLD‐PPA: Frontotemporal Lobar Degeneration with Primary Progressive Aphasia)).

We chose the temporal cortex for the PART and AD cases because NFTs, a prominent pathological hallmark of AD, are detectable in this region at the beginning of intermediate AD stages (Braak stages 3–4). As for control samples, we used the frontal temporal cortex based on case and tissue availability. The tissue samples were sectioned to a 6 µm thickness as we determined it to be the optimal thickness for visualizing tau aggregates with minimal background (see Experimental Section). Before immunostaining, samples were treated with Xylene and Ethanol to deparaffinize and rehydrate the tissues. Afterward, we performed a heat‐induced antigen retrieval, followed by immunostaining with phospho‐specific primary antibodies (see Experimental Section). To reduce background and optical aberrations, we incorporated a tissue clearing step with 2,2‐thiodiethanol (see Experimental Section).

To assess tissue integrity, we stained the sections with antibodies against MAP2 and DAPI to visualize neuronal processes and nuclei, respectively (Figure S2, Supporting Information). Consistent with cytoskeletal disruption associated with neurodegeneration, MAP2 staining appeared weak and fragmented in AD tissue. Nonetheless, neuronal cell bodies and processes were readily detectable in all tissues, confirming overall preservation of tissue integrity. For immunostaining of tau aggregates, we used commercially available and previously validated^[^ ^48^ ^]^ phospho‐specific primary antibodies AT8, AT270, and AT180, targeting hyperphosphorylated tau residues p‐S202/T205, p‐T181, and p‐T231, respectively (Figure S1A, Supporting Information; **Tables** **2** and 3 in Experimental Section). To validate the specificity of selected p‐tau antibodies and characterize the histological environment of tau aggregates across the 12 cases, we performed immunohistochemistry (IHC) and immunofluorescence (IF) staining, followed by widefield and confocal imaging, respectively. As expected, IHC and IF images of tau‐pathology free controls showed no detectable p‐tau staining (**Figures** S3 and S4, Supporting Information). In contrast, IHC and IF images for PART (Braak 1‐3) and AD (Braak 4‐6) samples showed a progressively increasing staining of p‐tau, as well as tau aggregate burden (**Figures** S3 and S4, Supporting Information). In AD (Braak 6) samples, we observed staining patterns morphologically consistent with neurofibrillary tangles (NFTs) across imaging modalities (Figure S5A, Supporting Information). To rule out that sample treatment, including heat denaturation and antigen retrieval, influences aggregate size by breaking up large aggregates, we performed a control experiment in which heat denaturation and antigen retrieval were omitted, followed by immunofluorescence labeling and confocal imaging using the AT8 antibody in advanced AD tissue (where tau pathology is most abundant). These experiments revealed that tau aggregates were comparable in size between the treated and untreated samples (Figure S5B, Supporting Information). Together, these results demonstrate that sample treatment does not affect tau aggregates.

**Table 2:** List of primary antibodies.

Primary antibodies	Specificity (Target)	Host species	Catalog number	Lot number	Vendor
PTh231	p‐T231	Rabbit (monoclonal)	SAB4504563	117247	Sigma‐Aldrich
AT180	p‐T231	Mouse (monoclonal)	MN1040	ZH4399983	Invitrogen
AT270	p‐T181	Mouse (monoclonal)	MN1050	ZB4164362	Invitrogen
D9F4G	p‐T181	Rabbit (monoclonal)	12885	8	Cell signaling
AT8	p‐S202/T205	Mouse (monoclonal)	MN1020	Z54507412	Fisher scientific
T22	Tau Oligomers	Rabbit (polyclonal)	ABN454	4104564	Sigma‐Aldrich
MAP2	MAP2	Rabbit (monoclonal)	EPR19691	1044743‐12	Abcam

**Table 3:** List of secondary antibodies and imaging oligos.

Secondary antibodies & Imager oligos	Specificity (Target)	Catalog number	Lot number	Vendor
Docking Site 1	Mouse IgG	N/A	2010003	Massive photonics
Docking Site 2	Rabbit IgG	N/A	2020003	Massive photonics
Imager Oligo 1‐ATTO655	Docking Site 1	N/A	2010003	Massive photonics
Imager Oligo 2‐Cy3b	Docking Site 2	N/A	2020003	Massive photonics
Alexa‐Fluor 546	Mouse IgG (H+L)	A21206	2541645	Invitrogen
Alexa‐Fluor 488	Rabbit IgG (H+L)	A10036	2482939	Invitrogen
Horse, Biotinylated	Mouse IgG (H+L)	BA‐2000	ZJ0725	Vector laboratories
Goat, Biotinylated	Rabbit IgG (H+L)	BA‐1000	ZK1020	Vector laboratories

### 2.2 Single‐Color Super‐Resolution Microscopy Pipeline for Visualizing Tau Aggregates in Human Postmortem Brain Tissues

**Figure 1:**
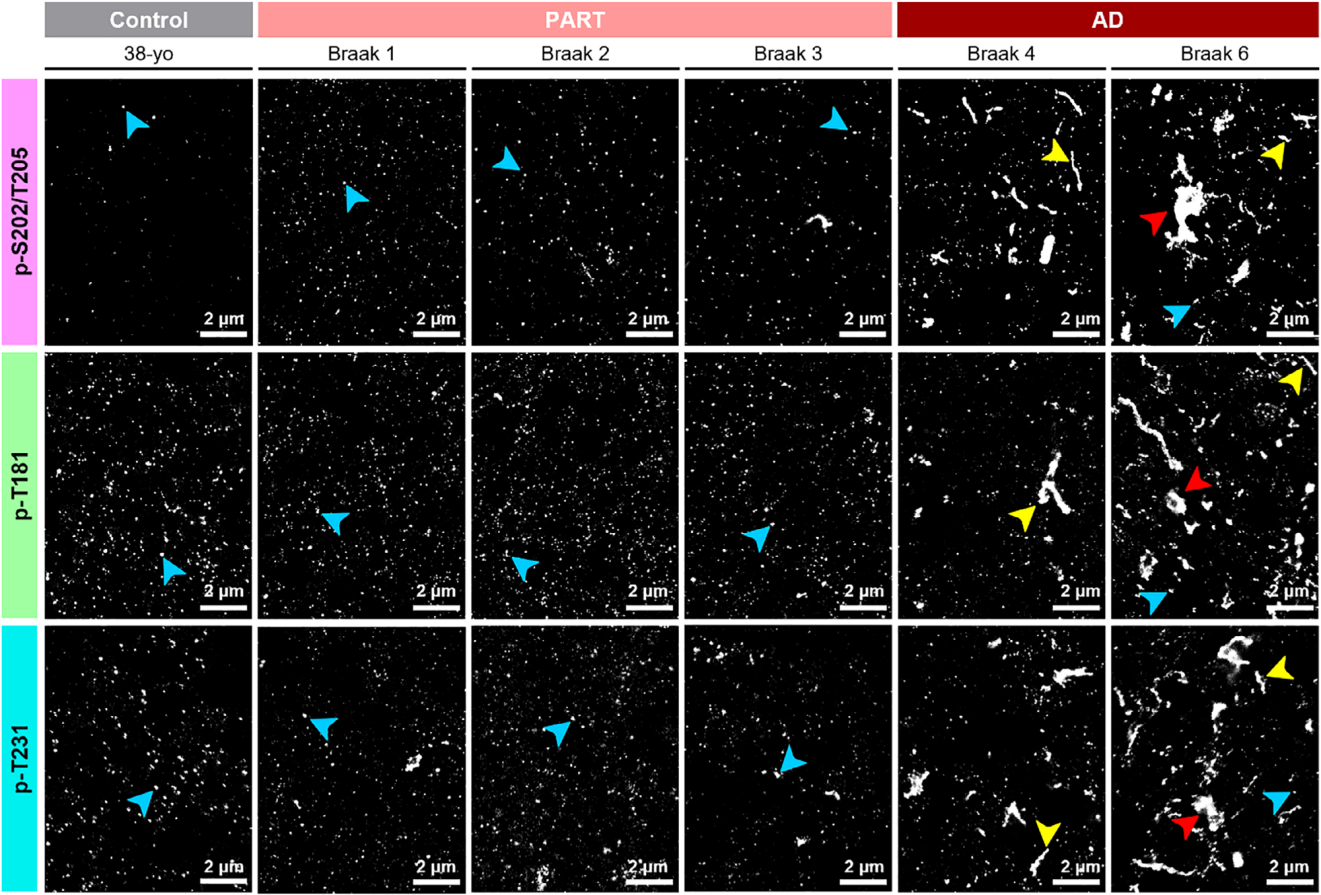
Single‐color DNA‐PAINT images of tau aggregates in human postmortem brain tissues. Representative single‐color DNA‐PAINT images of hyperphosphorylated tau in tissue sections from 38‐yo control, PART (Braak 1‐3), and AD (Braak 4 and 6) immunolabeled with AT8 (p‐202/s205), AT270 (p‐T181) and AT180 (p‐T231) primary antibodies. Images reveal the presence of an array of large aggregates, such as amorphous aggregates (red arrowhead), fibrillar aggregates (yellow arrowhead), and nano‐sized, punctate tau aggregates (blue arrowheads). AT8 targets (p‐S202/T205) were imaged with imager probe 1‐ATTO655. AT270 targets (p‐T181) were imaged with imager probe 2‐Cy3b. AT180 targets (p‐T231) were imaged with imager probe 2‐Cy3b. Acquisition parameters: 100x/1.45NA oil immersion objective, 10 mW 647‐ and 2.5 mW 561 nm lasers, gain‐not applicable, 100 ms exposure time.

To visualize tau aggregates at higher spatial resolution, we leveraged DNA‐PAINT super‐resolution microscopy. For this, we used secondary antibodies conjugated to unique orthogonal DNA‐docking oligos, facilitating DNA‐PAINT imaging (see Experimental Section and Figure S1A,B, Supporting Information). Single‐color super‐resolution DNA‐PAINT images revealed tau proteins hyperphosphorylated at residues p‐S202/T205, p‐T181, and p‐T231 within tau aggregates (**Figure** **1**). These aggregates appeared to increase in abundance, size, and morphological complexity at each examined Braak stage, mirroring IHC and IF images (Figure 1; **Figures** S3 and S4, Supporting Information). Specifically, images of AD (Braak 4 and 6) samples contained a wide variety of aggregates, including nanometric, punctate aggregates (Figure 1, **cyan arrows**) as well as larger aggregates such as linear fibrillar structures (Figure 1, **yellow arrows**) and amorphous aggregates resembling NFTs (Figure 1, **red arrows**). The larger amorphous aggregates were mostly absent in images from PART cases (Braak 1‐3) and controls, while some nanometric, punctate aggregates were visible in these samples (Figure 1, **cyan arrows**). To confirm that the nanometric aggregates we observed corresponded to tau aggregates rather than background and noise, we performed negative control experiments by immunolabeling control (38‐yo), PART (Braak 2), and AD (Braak 6) cases with only secondary DNA‐PAINT antibodies without primary phospho‐tau antibodies (Figure S6, Supporting Information). These negative controls enabled us to assess the background signal arising from non‐specific secondary antibody labeling and off‐target interactions from imager oligos in DNA‐PAINT images. Images of the negative controls exhibited significantly fewer detected localizations compared to positive samples, and the background puncta were smaller in size than the tau aggregates visualized in positive samples (compare Figure 1 to Figure S6, Supporting Information).

To quantify the abundance, size, and morphology of p‐tau aggregates in DNA‐PAINT images, we developed a quantitative analysis pipeline (Figure S7, Supporting Information). After acquisition and rendering of point‐cloud localizations we applied Voronoi Tessellation,^[^ ^44^, ^49^ ^]^ a commonly utilized segmentation strategy for super‐resolution microscopy, to segment individual tau aggregates in images from all samples, including the negative controls (see Experimental Section; Figure S7, Supporting Information, **steps 1 and 2**). To remove the background and noise from the images, we plotted the area distribution of the segmented objects in the images of negative controls (Figure S7, step 2 and Figure S8, Supporting Information). To remove background/noise from images of the positive samples, we applied an area threshold (indicated by the dashed line and blue shaded area in the histograms in Figure S7, **step 2** and Figure S8, **top and middle panels**, Supporting Information). This step effectively excluded the majority (>97%) of background/noise structures corresponding to those segmented in the negative controls (Figure S7, Supporting Information, **step 2 bottom images**).

After applying this filter, we reassessed the area distribution of tau aggregates in the filtered images from controls (38‐yo and 60‐yo), PART (Braak 1‐3), and AD (Braak 4‐6) (Figure S7, **step 3** and Figure S8, **bottom panels**, Supporting Information). We observed a gradual shift in the area distribution of tau aggregates toward larger areas across the Braak stages (Figure S7, **step 3** and Figure S8, **bottom panels**, Supporting Information), reflecting the progressive aggregation process in which tau oligomers seed the formation of fibrils and larger amorphous aggregates, including NFTs. Utilizing these area distributions, we classified tau aggregates into three classes: nano‐aggregates (average of ≈90 nm diameter), intermediate‐aggregates (average of ≈500 nm major axis), and micro‐aggregates (average of ≈1.6 µm major axis) (Figure S7, **step 3** and Figure S8, **bottom panels**, Supporting Information). Further visual inspection of tau aggregates within these classes confirmed a progression in size and morphology, starting with small, punctate nano‐aggregates, evolving to larger, more complex aggregates (Figure S7, Supporting Information **step 3 bottom images**).

Overall, we successfully established a quantitative super‐resolution microscopy pipeline that effectively identifies p‐tau enriched tau aggregates, ranging from nano‐ to micron‐sized, in PART (Braak 1‐3) and AD (Braak 4‐6) samples.

### 2.3 p‐T231 and p‐T181 Nano‐Aggregates are Present in Tau‐Pathology Free Controls

**Figure 2:**
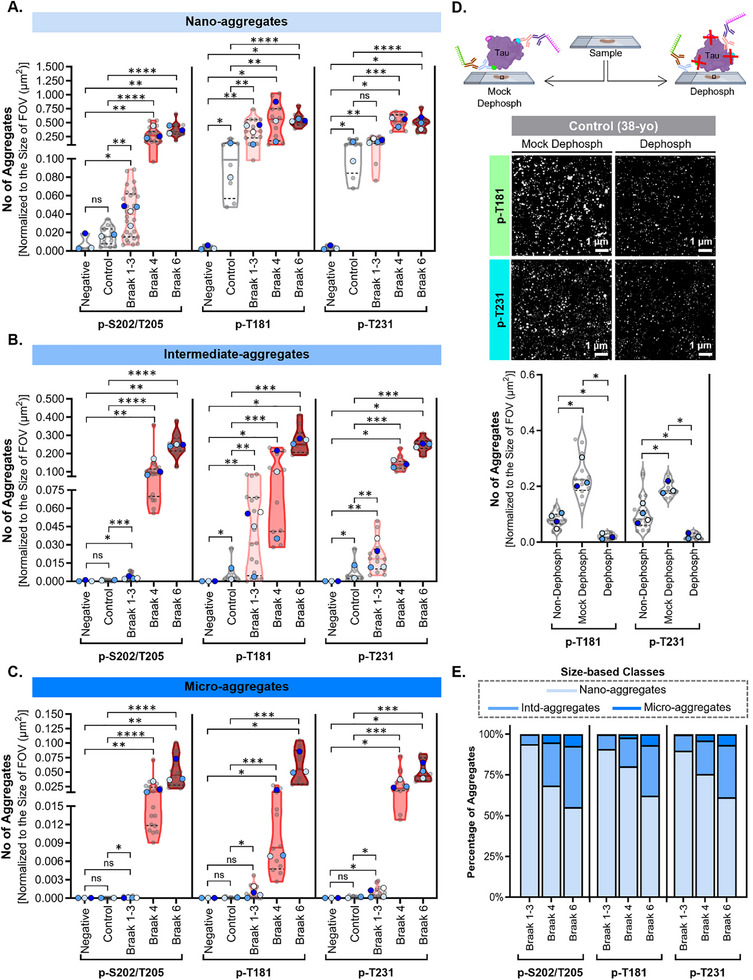
p‐tau nanoaggregates are the predominant tau aggregates across cases, and p‐T231 and p‐T181 nano‐aggregates are present in tau‐pathology free controls. Sample Size – (A‐C)) p‐S202/T205: Control (38‐yo and 60‐yo) N = 16 tissue sections (8 per case), *n* = 64 collective fields of view; PART (Braak 1‐3) N = 28 tissue sections (8 for Braak 1 case, 12 for Braak 2 cases, and 8 for Braak 3 case), *n* = 112 collective fields of view; AD (Braak 4) N = 20 tissue sections (4 for case A, 8 for cases B‐C), *n* = 79 collective fields of view; AD (Braak 6) N = 18 tissue sections (4 for case A, 8 for case B, 6 for case C), *n* = 78 collective fields of view. p‐T181: Control (38‐ and 60‐yo) N = 8 tissue sections (4 per case), *n* = 32 collective fields of view; PART (Braak 1‐3) N = 16 tissue sections from (4 per case), *n* = 64 collective fields of view; AD (Braak 4) N = 12 tissue sections (4 per case), *n* = 51 collective fields of view; AD (Braak 6) N = 11 tissue sections (4 for cases A,B, 3 for case C), *n* = 46 collective fields of view. p‐T231: Control (38‐ and 60‐yo) N = 8 tissue sections (4 per case), *n* = 32 collective fields of view; PART (Braak 1‐3) N = 16 tissue sections (4 per case), *n* = 65 collective fields of view; AD (Braak 4) N = 12 tissue sections (4 per case), *n* = 49 collective fields of view; AD (Braak 6) N = 11 tissue sections (4 for cases A‐B, 3 for case C), *n* = 45 collective fields of view. Sample Size – (A‐C)) Negative Controls for p‐S202/T205, p‐T181, and p‐T231: 38‐yo, PART (Braak 2), AD (Braak 6) N = 3 tissue sections (1 per case), n = 4 fields of view per section. Sample Size – (D)) Non‐dephosph (38‐yo): N = 4 tissue sections, n=16 collective fields of view; Mock dephosph (38‐yo): N = 3 tissue sections, *n* = 12 fields of view; Dephospho (38‐yo): N = 3 tissue section, *n* = 12 fields of view. Statistical Tests – (A–C) An unpaired two‐sided rank sum test was performed using the means per biological replicates (light grey dots). A *p* value < 0.05 was taken as statically significant. *P* values: ns (>0.05), * (0.05 – 0.01), ** (0.001 – 0.01), *** (0.0001 – 0.001), **** (< 0.0001). Statistical Tests – (D)) An unpaired one‐sided rank sum test was performed using the means per biological replicates (large colored dots) to test the hypothesis that dephosphorylation leads to a decrease in the number of phospho‐tau nano‐aggregates. A *p* value < 0.05 was taken as statically significant. *P* values: ns (>0.05), * (0.05 – 0.01), ** (0.001 – 0.01), *** (0.0001 – 0.001), **** (< 0.0001). Acquisition parameters: 100x/1.45NA oil immersion objective, 2.5 mW 561‐nm lasers, gain‐not applicable, 100‐ms exposure time. A–C) Quantification of the number of tau A) nano‐ B) intermediate‐ and C) micro‐aggregates per unit area in negative controls, which include 38‐yo, PART (Braak 2), AD (Braak 6), and samples corresponding to control (light grey: 38‐yo and 60‐yo), PART (light pink: Braak 1‐3), and AD (dark pink: Braak 4, dark red: Braak 6). Violin plots show the distribution of the mean number of tau aggregates per replicate. Solid line indicates median and dashed lines indicate the quartile percentiles (25th and 75th). Small grey dots represent the mean number of tau aggregates per replicate (tissue section) and larger dots represent the mean number of tau aggregates per case, starting with case A (light blue), case B (periwinkle blue), case C (dark blue), and case D (white). D) Representative diagram of dephosphorylation assay to validate AT270 (p‐T181) and AT180 (p‐T231) primary antibody specificity. Tissue sections from control (38‐yo) were treated with dephosphorylating enzyme (dephosph). Images show representative examples of mock dephosphorylated (mock dephosph) and dephosphorylated (dephosph) 38‐yo control samples labeled for p‐T181 and p‐T231. Violin plots show the distribution of the mean number of tau aggregates per unit area. Solid line indicates median, and dashed lines indicate the quartile percentiles (25th and 75th). Small grey dots represent an individual field of view and large colored dots indicate each biological replicate (independent tissue sections mock dephosphorylated or dephosphorylated). The violin plot for the non‐dephosphorylated (non‐dephosph) samples has been reproduced from (A) including only the 38‐yo control. E) Percentages of p‐S202/T205, p‐T181, and p‐T231 positive tau aggregates were calculated from the data presented in (A–C) using the mean number of aggregates per case. Stacked bar plot shows the mean percentage (light blue=nano‐aggregates, periwinkle blue=intermediate‐aggregates, and dark blue=micro‐aggregates).

**Figure 3:**
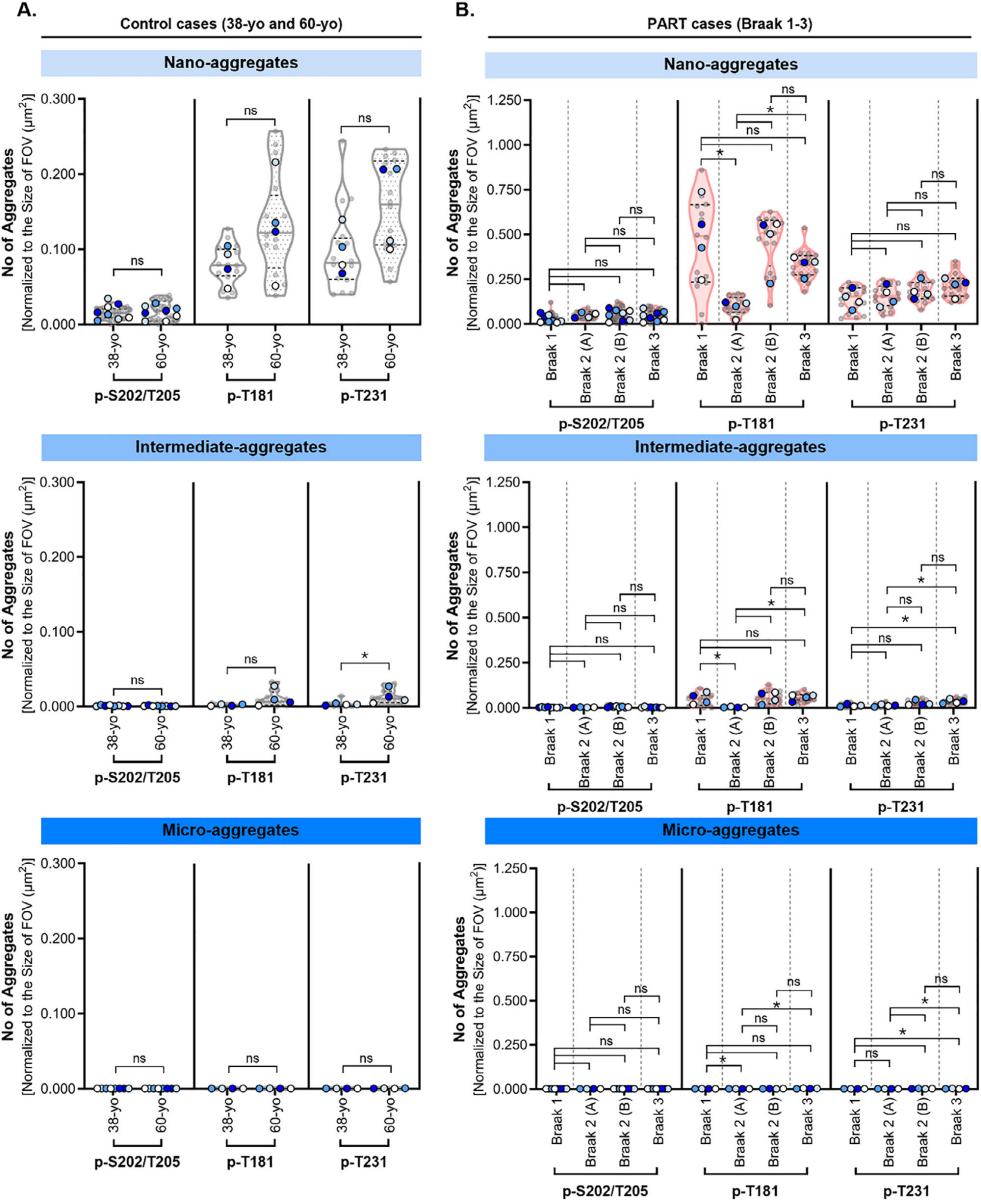
Number of p‐tau nano‐aggregates from control (38‐ and 60‐yo) and PART (Braak 1‐3) images does not vary across cases examined. Sample Size – (A,B) p‐S202/T205, p‐T181, and p‐T231: p‐S202/T205: Control: (38‐yo) N = 8 tissue sections, *n* = 32 fields of view; (60‐yo) N = 8 tissue sections, n=32 fields of view; PART: (Braak 1) N = 8 tissue sections, *n* = 31 fields of view, (Braak 2‐patient A) N=4 tissue sections, *n* = 16 fields of view; (Braak 2‐patient B) N = 8 tissue sections, *n* = 32 fields of view; (Braak 3) N = 8 tissue sections, *n* = 31 fields of view. p‐T181 Control: (38‐yo) N = 4 tissue sections, *n* = 16 fields of view; (60‐yo) N = 4 tissue sections, *n* = 16 fields of view; PART: (Braak 1) N = 4 tissue sections, *n* = 15 fields of view, (Braak 2‐patient A) N = 4 tissue sections, *n* = 17 fields of view; (Braak 2‐patient B) *N* = 4 tissue sections, *n* = 16 fields of view; (Braak 3) N = 4 tissue sections, *n* = 16 fields of view. p‐T231 Control: (38‐yo) N = 4 tissue sections, *n* = 16 fields of view; (60‐yo) N = 4 tissue sections, *n* = 16 fields of view; PART: (Braak 1) N = 4 tissue sections, *n* = 16 fields of view, (Braak 2‐patient A) N = 4 tissue sections, *n* = 16 fields of view; (Braak 2‐patient B) N = 4 tissue sections, *n* = 16 fields of view; (Braak 3) N = 4 tissue sections, *n* = 17 fields of view. Statistical Tests – (A,B) An unpaired two‐sided rank sum test was performed using the means per biological replicate (blue‐colored dots). A *p* value <0.05 was taken as statically significant. *P* values: ns (>0.05), * (0.05–0.01), ** (0.001–0.01), *** (0.0001–0.001), **** (< 0.0001). A,B) Quantification of the number of tau nano‐, intermediate‐, and micro‐aggregates per unit area in control (light grey: 38‐yo and 60‐yo) and PART (light pink: Braak 1–3) cases comparing variability between different aged controls and different Braak stages of PART. From top to bottom) Violin plots show the distribution of the mean number of tau aggregates. Solid line indicates median and dashed lines indicate the quartile percentiles (25th and 75th). Small grey dots represent the mean number of aggregates per field of view, and larger dots represent the mean number of aggregates per replicate (tissue section), starting with replicate 1 (light blue), replicate 2 (periwinkle blue), replicate 3 (dark blue), and replicate 4 (white).

To determine whether the abundance of nano‐, intermediate‐, and micro‐aggregates varies across AD stages and if this is correlated to the hyperphosphorylation of specific residues, we quantified the number of tau aggregates per unit area across the tissue samples (**Figure** **2**A–C). As expected, almost no tau aggregates of any size‐based class were found in images from secondary antibody only negative control samples (Figure 2A–C). As the number of p‐S202/T205, p‐T181, and p‐T231 positive aggregates did not significantly differ between different control cases (38‐yo and 60‐yo) (**Figure** **3**A) and PART cases (Braak 1‐3) (Figure 3B), we pooled the data corresponding to control (38‐yo and 60‐yo) and PART (Braak 1‐3) for simplicity, although future studies with larger patient cohorts are needed to more rigorously assess and validate inter‐sample variability. Surprisingly, a significant increase in the number of p‐T181 and p‐T231 positive nano‐aggregates was observed in tau‐pathology free control (38‐yo and 60‐yo) and PART (Braak 1‐3) cases when compared to negative controls (Figure 2A). In contrast, p‐S202/T205 positive nano‐, intermediate‐, and micro‐aggregates were absent in control cases and detected at low levels in PART (Braak 1‐3) cases (**Figures** 2A–C).

To ensure that p‐T181 and p‐T231 positive nano‐aggregates detected in control cases (38‐yo and 60‐yo) correspond to real signal as opposed to non‐specific primary antibody binding, we carried out additional control experiments, in which we treated tissue samples from the 38‐yo control case with a dephosphorylating enzyme (dephosph) to remove phosphorylated epitopes or mock dephosphorylated (mock‐dephosph) in the absence of the enzyme (**Figure** 2D). As an additional validation step, we performed the dephosphorylation protocol with tissue samples from an AD (Braak 6) case, which resulted in a drastic reduction of p‐tau signal (p‐T181 and p‐T231) compared to mock‐dephosph controls, as confirmed by IHC and IF imaging (Figure S9A,B, Supporting Information). Analysis of DNA‐PAINT images from mock‐dephosphorylated samples of the control case (38‐year‐old) revealed an apparent increase in p‐T181 and p‐T231 signals compared to untreated samples, likely reflecting enhanced autofluorescence and background following the mock treatment. Nevertheless, the number of nano‐aggregates was drastically reduced in the dephosphorylated samples relative to both mock‐treated and untreated controls, strongly suggesting detection of real signal as opposed to non‐specific antibody binding (Figure 2D). Hence, our findings reveal that by using DNA‐PAINT, it is possible to detect nano‐aggregates containing p‐T181 and p‐T231 modified tau in brain tissues devoid of any tau pathology, likely representing physiological tau oligomers.

The abundance of nano‐aggregates containing p‐tau, particularly at residues S202/T205, significantly increased in AD (Braak 4 and 6) compared to controls and PART (Figure 2A). Although the abundance of nano‐aggregates between intermediate AD (Braak 4) and advanced AD (Braak 6) did not change significantly (Figure 2A), a marked increase in the burden of intermediate‐ and micro‐aggregates, particularly those positive for p‐T181, was observed for advanced AD (Braak 6) in comparison to intermediate AD (Braak 4) (Figure 2B,C). These results suggest that the appearance of p‐T181‐positive tau within large aggregates may be a hallmark of more advanced AD.

We further determined the percentage of the different classes (nano‐, intermediate‐ and micro‐) of tau aggregates (Figure 2E). We found that nano‐aggregates constitute the majority of aggregates in PART (Braak 1‐3) and intermediate AD (Braak 4) (over 90% in PART and over 70% in AD Braak 4). Notably, even for advanced AD (Braak 6), nano‐aggregates account for over 50% of all p‐tau aggregates. This pattern persisted across all visualized p‐tau residues, suggesting that nano‐aggregates in AD (Braak 4 and 6) samples contain tau proteins hyperphosphorylated at “early” (p‐T231, p‐T181) and “late” (p‐S202/T205) residues, whereas PART (Braak 1–3) and control (38‐yo and 60‐yo) samples mainly contain nano‐aggregates hyperphosphorylated at “early” (p‐T231, p‐T181) residues. Overall, our data highlight that the large, micron‐sized aggregates detectable by conventional IF or IHC represent only a minor fraction of the total p‐tau aggregate burden. Hence, most aggregates remain smaller than the detection and resolution limit of these imaging techniques, underscoring the predominance of nano‐aggregates in AD pathology.

To further characterize the nature of these prevalent nano‐aggregates, we performed DNA‐PAINT imaging of AD (Braak 6) sample immunolabeled with tau oligomer‐specific antibody, T22 (Figure S9C, Supporting Information).^[^ ^36^ ^]^ This analysis revealed that T22‐positive nanometric aggregates are comparable in size and abundance to those detected with phospho‐tau antibodies (Figure S9C–E, Supporting Information), supporting the interpretation that a subset of these nano‐aggregates is likely tau oligomers, while their broad size range likely reflects a heterogeneous spectrum of tau assemblies extending from soluble oligomers to larger, insoluble protofibrils.

### 2.4 Distinct p‐Tau Nano‐Aggregate Morphology Differentiates AD from PART and Controls

**Figure 4:**
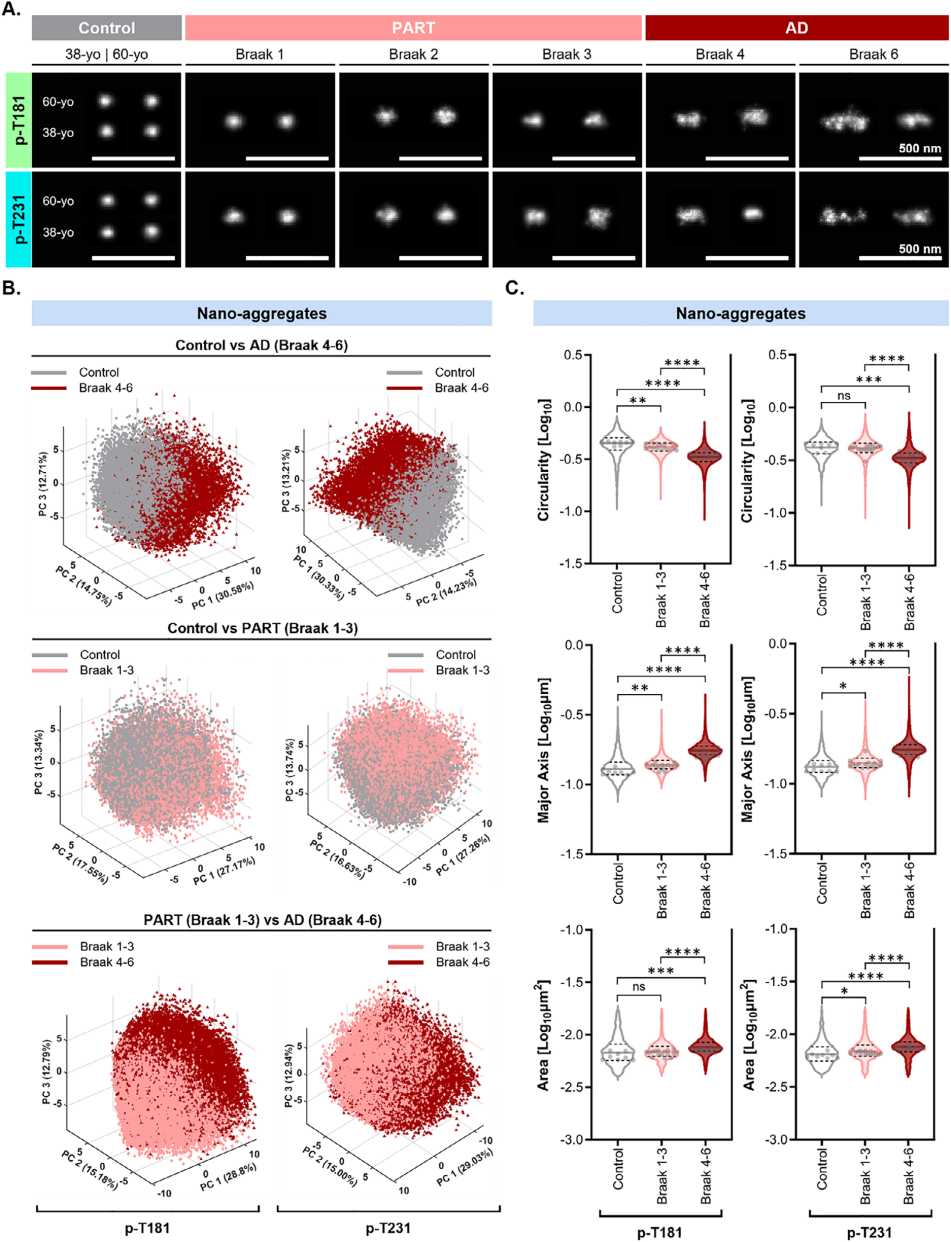
Morphological analysis of p‐tau nano‐aggregates reveals distinct characteristics between control, PART, and AD. Sample Size – A–C) p‐T181 Control (38‐yo and 60‐yo) N = 8 tissue sections (4 per case), *n* = 32 collective fields of view; PART (Braak 1‐3) N = 16 tissue sections (4 per case), *n* = 64 collective fields of view; AD (Braak 4) *N* = 12 tissue sections (4 per case), *n* = 51 collective fields of view; AD (Braak 6) *N* = 11 tissue sections (4 for cases A‐B, 3 for case C), *n* = 46 collective fields of view. p‐T231: Control (38‐yo and 60‐yo) *N* = 8 tissue sections (4 per case), *n* = 32 collective fields of view; PART (Braak 1‐3) *N* = 16 tissue sections (4 per case), *n* = 65 collective fields of view; AD (Braak 4) N=12 tissue sections (4 per case), *n* = 49 collective fields of view; AD (Braak 6) *N* = 11 tissue sections (4 for cases A‐B, 3 for case C), *n* = 45 collective fields of view. Statistical Tests – An unpaired two‐sided rank sum test was performed using the means per replicate (light grey dots). A *p* value < 0.05 was taken as statistically significant. *P* values: ns (>0.05), * (0.05 – 0.01), ** (0.001–0.01), *** (0.0001–0.001), **** (< 0.0001). A) Representative examples of p‐T181 and p‐T231 positive nano‐aggregates found in images from control (38‐yo and 60‐yo), PART (Braak 1‐3), and AD (Braak 4 and 6) samples. B) Principal Component Analysis (PCA) plots showing the first three principal components (PCs) for p‐T181 and p‐T231 positive nano‐aggregates in control (light grey: 38‐yo and 60‐yo), PART (light pink: Braak 1‐3), and AD (dark pink: Braak 4 and 6). C) Violin plots showing the distribution of p‐T181 and p‐T231 positive nano‐aggregates’ circularity (log_10_), major axis (log_10_µm), and area (log_10_µm^2^) in control (light grey: 38‐yo and 60‐yo), PART (light pink: Braak 1‐3), and AD (dark pink: Braak 4 and 6). Solid line indicates median, and the dashed lines indicate the quartile percentiles (25th and 75th). Small grey dots represent the mean value (circularity, major axis, and area) per biological replicate (tissue section).

**Figure 5:**
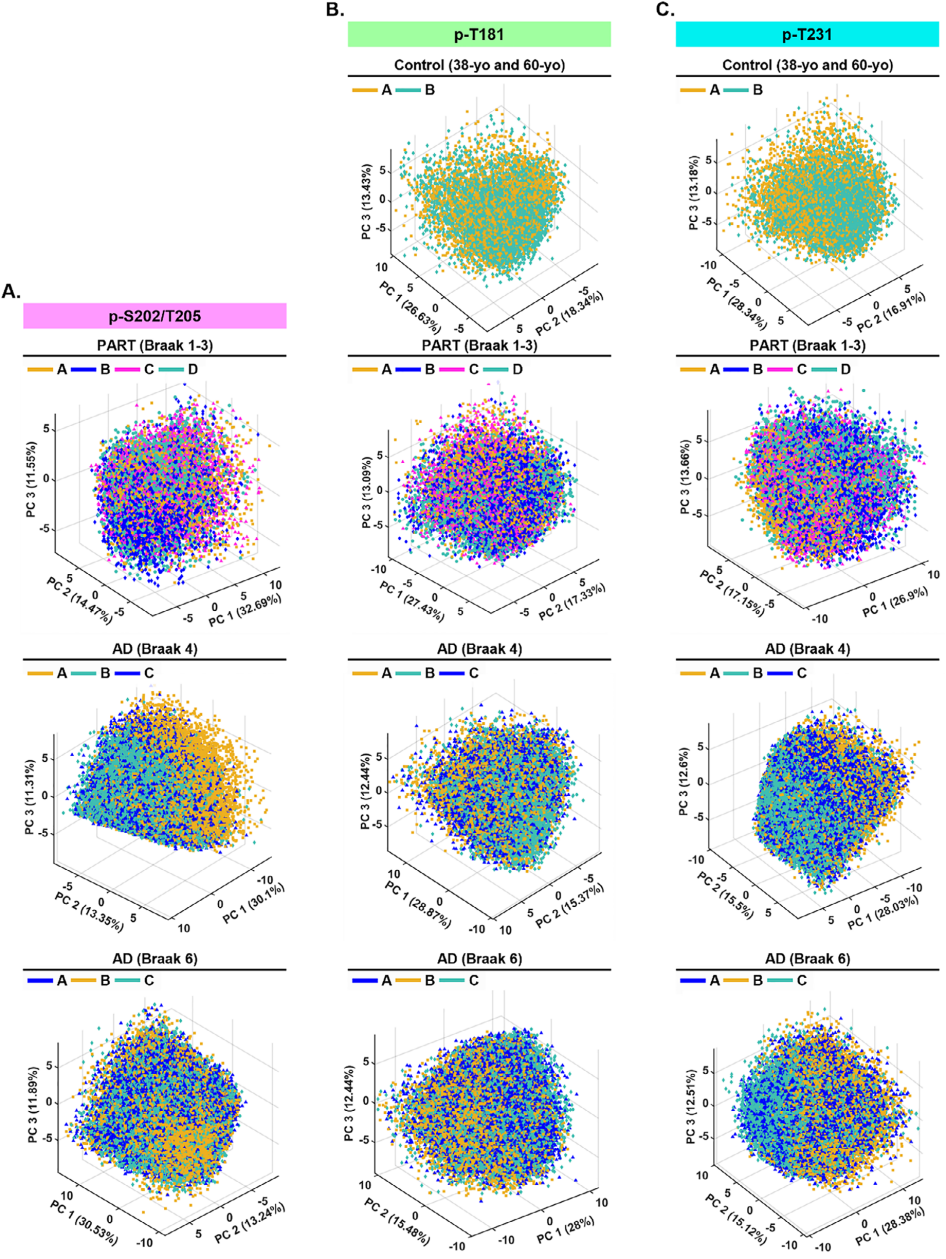
PCA plots show a high level of morphological overlap in tau nano‐aggregates between individual cases. Sample Size – A–C) p‐S202/T205, p‐T181, and p‐T231: p‐S202/T205: PART (Braak 1‐3) N = 28 tissue sections (8 for Braak 1 case, 12 for Braak 2 cases, and 8 for Braak 3 case), *n* = 112 collective fields of view; AD (Braak 4) *N* = 20 tissue sections (4 for case A, 8 for cases B‐C), *n* = 79 collective fields of view; AD (Braak 6) *N* = 18 tissue sections (4 for case A, 8 for case B, 6 for case C), *n* = 78 collective fields of view. p‐T181: Control (38‐ and 60‐yo) *N* = 8 tissue sections (4 per case), *n* = 32 collective fields of view; PART (Braak 1‐3) N = 16 tissue sections (4 per case), *n* = 64 collective fields of view; AD (Braak 4) N = 12 tissue sections (4 per case), *n* = 51 collective fields of view; AD (Braak 6) N = 11 tissue sections (4 for cases A‐B, 3 for case C), *n* = 46 collective fields of view. p‐T231: Control (38‐ and 60‐yo) N = 8 tissue sections (4 per case), *n* = 32 collective fields of view; PART (Braak 1‐3) N = 16 tissue sections (4 per case), *n* = 64 collective fields of view; AD (Braak 4) N = 12 tissue sections (4 per case), *n* = 49 collective fields of view; AD (Braak 6) N = 11 tissue sections (4 for cases A‐B, 3 for case C), *n* = 45 collective fields of view. A–C) From top to bottom: Principal Component Analysis (PCA) plots showing the first three principal components for p‐T202/S205 nano‐aggregates A), p‐T181 nano‐aggregates B), and pT231 nano‐aggregates C). First Row: control cases (yellow = 38‐yo and green= 60‐yo). Second Row: PART cases (yellow = Braak 1, blue = Braak 2 Case A, magenta = Braak 2 Case B, and green = Braak 3); Third Row: Intermediate AD (Braak 4) cases (yellow = Case A, green = Case B, and blue = Case C); Fourth Row: Advanced AD (Braak 6) cases (blue = Case A, yellow = Case B, and green = Case C). Control cases lack plots for p‐T202/s205 as these nano‐aggregates are absent in controls.

Given the high prevalence of tau nano‐aggregates across cases, we focused on characterizing this class of aggregates further to determine if their morphology varies across Braak stages, and determine if “physiological” nano‐aggregates found in control cases are distinct from those found in PART or AD. To this end, we applied our recently developed algorithm called ECLiPSE (Enhanced Classification of Localized Point‐clouds by Shape Extraction)^[^ ^50^ ^]^ to extract 67 distinct morphological features, including geometric, boundary, skeleton‐based parameters, for each p‐tau nano‐aggregate. Using Principal Component Analysis (PCA), we investigated whether p‐T231, p‐T181, and p‐T202/S205 positive nano‐aggregates showed distinct clustering in terms of morphology among control, PART, and AD cases (**Figure** **4**A,B). We first assessed patient‐to‐patient variability by comparing the two control cases (38‐yo and 60‐yo), four PART cases (Braak 1‐3), three intermediate AD cases (Braak 4), and three advanced AD cases (Braak 6). PCA plots revealed substantial overlap among the examined cases, indicating minimal inter‐individual morphological variability within each group (**Figure** **5**). We therefore combined the individual cases belonging to each category together for the subsequent disease stage comparisons for simplicity.

We next compared p‐T181 and p‐T231 nano‐aggregates across Braak stages since these modifications are observed under physiological and pathological conditions. Notably, intermediate (Braak 4) and advanced (Braak 6) AD cases showed substantial overlap in PCA space (Figure S10A, Supporting Information). As a result, we further combined these two stages into a single AD group for subsequent comparisons with PART (Braak 1–3) and control cases (38‐yo and 60‐yo) for simplicity. Interestingly, nano‐aggregates from control and AD samples formed separated clusters in the PCA space with the lowest amount of overlap (46%) (**Figure** 4B, **top plots**). p‐T181 and p‐T231‐positive PART nano‐aggregates exhibited partial overlap with control (80%) (Figure 4B, **middle plots**) and AD (67%) cases (Figure 4B, **bottom plots**), with the greatest degree of overlap observed between PART and control. Similarly, p‐S202/T205 positive nano‐aggregates partially overlapped between PART and AD (Figure S10B, Supporting Information). These results suggest that PART nano‐aggregates represent an intermediate state in the progression from physiological to pathological forms, and that morphological evolution of nano‐aggregates plateaus at later stages of AD. Further analysis of specific morphological features (area, circularity, and major axis) showed that nano‐aggregates in AD were less circular, with a longer major axis and a larger area compared to those in PART or control cases (Figure 4C; Figure S10B, Supporting Information).

Together, these findings reveal that tau nano‐aggregates exhibit distinct morphological characteristics that clearly distinguish AD from non‐pathological states, while remaining relatively stable across intermediate and late AD.

### 2.5 Multicolor DNA‐PAINT Imaging Pipeline for Characterizing Multi‐Site Hyperphosphorylation Profiles of Tau Aggregates

**Figure 6:**
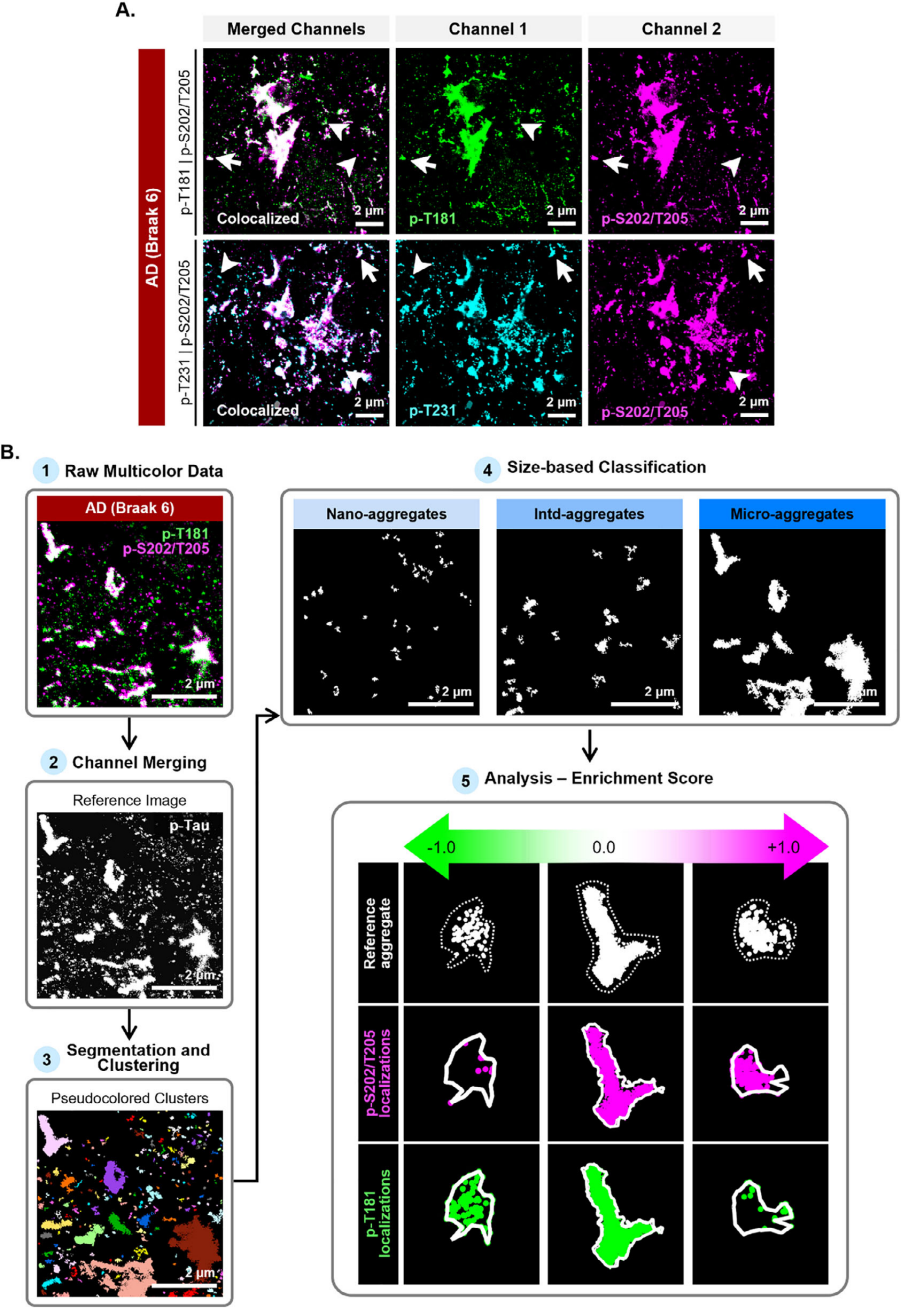
Multicolor DNA‐PAINT imaging and analysis pipeline enables the identification and evaluation of multiple hyperphosphorylated residues within a single‐tau aggregate. Sample Size – p‐S202/T205 and p‐T181: AD (Braak 6) N = 11 tissue sections (4 for case A‐B, 3 for case C), *n* = 43 collective fields of view; p‐S202/T205 and p‐T231: AD (Braak 6) N = 11 tissue sections (4 for case A‐B, 3 for case C), *n* = 44 collective fields of view. A) Representative dual‐color DNA‐PAINT images of AD (Braak 6) sections immunolabeled with combinations of primary antibodies AT8, AT270, and AT180 to identify p‐S202/T205 (magenta), p‐T181 (green), and p‐T231 (cyan) positive aggregates. Overlay reveals the presence of both targets within individual aggregates (white arrow) as well as aggregates with tau proteins predominantly modified at a single target (white arrowhead). Acquisition parameters) 100x/1.45NA oil immersion objective, 10 mW 647‐ and 2.5 mW 561‐nm lasers, gain‐not applicable, 100 ms exposure time. B) Dual‐color DNA‐PAINT image analysis pipeline. 1) Localizations for both targets are acquired following the pipeline described in Figure 1. Image shows the localizations for p‐T181 tau (green) and p‐S02/T205 tau (magenta). 2) The localizations from both targets are merged to generate a reference image (white image). 3) The reference image is clustered and segmented using Voronoi tessellation. The image shows pseudo‐colored segmented reference clusters, or tau aggregates. 4) After background filtering and quality control steps (see Figure S11, Supporting Information), segmented p‐tau aggregates are classified into the three size‐based classes (nano‐, intermediate‐, and micro‐aggregates). 5) To determine the relative abundance of each target within an individual tau aggregate, the number of localizations belonging to each target within the individual reference tau aggregates is computed and converted to an enrichment score, ranging from −1 to 1.

Our findings revealed that tau nano‐, intermediate‐, and micro‐aggregates in AD consist of tau proteins hyperphosphorylated at different residues. Tau is thought to undergo combinatorial hyperphosphorylation in disease, with modifications at certain residues, such as p‐T181, facilitating further hyperphosphorylation of other residues.^[^ ^51^ ^]^ Therefore, we sought to determine whether these phospho‐tau residues coexisted within the same tau aggregates in a combinatorial fashion or if they delineated separate tau aggregates. To address this question, we carried out dual‐color DNA‐PAINT super‐resolution microscopy employing combinations of p‐T181 and p‐S202/T205 or p‐T231 and p‐S202/T205 (**Figure** **6** **A**).

To quantitatively analyze dual‐color DNA‐PAINT images, we developed a co‐localization analysis pipeline (Figure 6B). First, we combined the two channels corresponding to the two modifications into a single reference channel and performed segmentation (Figure 6B, **steps 1‐3**). This ensured the segmentation of a unified set of tau aggregates from both channels, using consistent segmentation parameters. To ensure accurate segmentation, we implemented a quality control step in which segmented localizations were assigned to their respective color channels and assessed for high spatial overlap within each segmented object, consistent with the expectation that both modifications label the same tau aggregate if the overlap is high (Figure S11, Supporting Information).

Following segmentation and filtering, we once again classified tau aggregates into three classes: nano‐, intermediate‐, and micro‐aggregates, utilizing the area cut‐offs previously established (Figure 6B, **step 4**). In the final step, we divided the detected localizations corresponding to each reference tau aggregate back into their original two‐color channels and then determined the number of detected localizations for each p‐tau residue (**Figure** 6B, **step 5**). In DNA‐PAINT, the number of detected localizations scales linearly with the number of visualized targeted protein.^[^ ^52^ ^]^ Consequently, we calculated the ratio between the difference of the detected localizations for each residue and the sum of the detected localizations for each residue (see Experimental Sections) to derive a relative enrichment score for each modification (Figure 6B, **step 5**). A score of ‐1 or +1 corresponds to tau aggregates containing p‐tau at one (e.g., p‐T181) or the other (e.g., p‐T202/S205) modification, while a score of 0 designates tau aggregates with an equal number of detected localizations from each p‐tau modification (Figure 6B, **step 5**).

To rule out steric hindrance as a confounding factor in dual labeling, we demonstrated a positive correlation between the localizations of the two p‐tau modifications (Figure S12A–C, Supporting Information), whereas steric hindrance would be expected to produce a negative correlation. To further validate that steric hindrance wasn't an issue, we compared the number of detected localizations within tau aggregates in single‐color and dual‐color DNA‐PAINT images. If steric hindrance affected antibody binding, we would expect to see a decrease in the number of detected localizations in the dual‐color images compared to the single‐color images. However, we did not observe such a decrease (Figure S12D–F, Supporting Information).

Taken together, these findings strongly suggest that the antibodies can bind to their respective targets simultaneously without steric hindrance, and that dual‐color DNA‐PAINT imaging is a robust strategy for determining the relative amounts of hyperphosphorylated tau proteins within individual tau aggregates.

### 2.6 Nano‐Aggregates Exhibit Braak Stage‐Specific Hyperphosphorylation Patterns in Alzheimer's Disease

**Figure 7:**
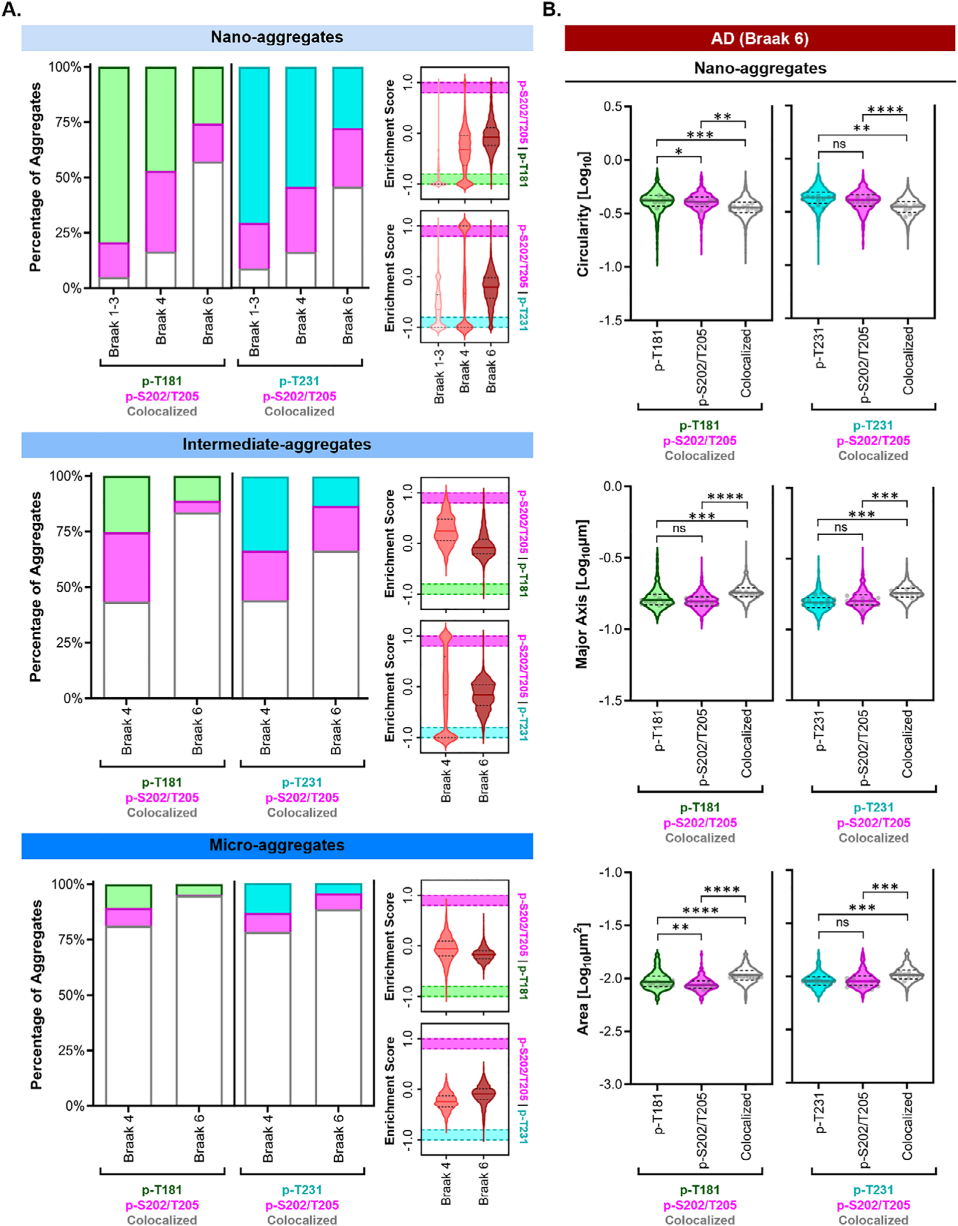
Nano‐aggregates are differentially hyperphosphorylated at advanced AD stages. Sample Size – A,B) p‐S202/T205 and p‐T181: PART (Braak 1‐3) N = 16 sections (4 per case), *n* = 63 collective fields of view; AD (Braak 4) N = 12 tissue sections (3 per case), *n* = 51 collective fields of view; AD (Braak 6) N = 11 tissue sections (4 for case A‐B, 3 for case C), *n* = 43 collective fields of view. p‐S202/T205 and p‐T231) PART (Braak 1‐3), *N* = 16 sections (4 per case), *n* = 65 collective fields of view; AD (Braak 4) N = 12 tissue sections (4 per case), *n* = 50 collective fields of view; AD (Braak 6) N = 11 tissue sections (4 for case A,B, 3 for case C), n=44 collective fields of view. Statistical Tests – An unpaired two‐sided rank sum test was performed using the means per replicate (light grey dots). A *p* value < 0.05 was taken as statically significant. *P* values: ns (>0.05), * (0.05–0.01), ** (0.001–0.01), *** (0.0001–0.001), **** (< 0.0001). A) From top to bottom: percentage of dually hyperphosphorylated (white) and singly hyperphosphorylated (magenta: p‐S202/T205, cyan: p‐T231, and green: p‐T181) tau nano‐, intermediate‐, and micro‐aggregates. The stacked bar plot shows the mean percentage of all tissue sections imaged. Violin plots show the distribution of enrichment scores of tau aggregates. B) Violin plots showing the distribution of singly modified tau: p‐T181 only (green), p‐S202/T205 only (magenta), p‐T231 only (cyan), and dually modified tau: p‐T181/p‐S202/T205 (white), p‐T231/p‐S202/T205 (white), AD (Braak 6) nano‐aggregates’ circularity (log10), major axis (log10µm), and area (log10µm^2^). Solid line indicates median, and the dashed lines indicate the quartile percentiles (25th and 75th). Small grey dots represent the mean value (circularity, major axis, and area) per biological replicate (tissue section).

The two‐color analysis pipeline enabled us to determine the extent of phosphorylation marks corresponding to distinct tau residues within individual tau aggregates (**Figure** **7**A), which is not possible using bulk biochemical analysis. Nano‐aggregates in PART (Braak 1–3) and intermediate AD (Braak 4) displayed a broader distribution of enrichment scores for both p‐T181 + p‐S202/T205 and p‐T231 + p‐S202/T205 combinations compared to advanced AD (Figure 7A, **violin plots**). These results indicate that tau nano‐aggregates in PART (Braak 1–3) and intermediate AD (Braak 4) exhibit heterogeneous phosphorylation patterns across examined residues. However, this variability diminished at advanced AD (Braak 6), where nano‐aggregates tended to contain more uniform levels of both p‐tau modifications (Figure 7A **, violin plots**). To further analyze the phosphorylation pattern of tau proteins within individual nano‐aggregates in a simpler manner, we applied cut‐offs to the enrichment scores (dashed lines in Figure 7A **, violin plots**). Scores below −0.8 and above 0.8 were categorized as “singly modified” tau aggregates, while scores between ‐0.8 and 0.8 were labeled as “dually modified” tau aggregates (Figure 7A **, stacked bar plots**). This analysis revealed that the majority of nano‐aggregates in PART (Braak 1–3) and intermediate AD (Braak 4) were enriched with tau proteins modified at only one of the p‐tau residues visualized (Figure 7A **, stacked bar plots**). While control experiments suggest that steric hindrance from antibody binding is minimal, we cannot entirely rule out the possibility that steric effects may impact the enrichment scores, leading to the underestimation of dually modified tau nano‐aggregates. In advanced AD (Braak 6), there was a substantial increase in the percentage of nano‐aggregates with tau proteins modified at both p‐tau residues, from 17% in AD (Braak 4) to 57% in AD (Braak 6) for p‐T181 + p‐S202/T205, and from 16% in AD (Braak 4) to 46% in AD (Braak 6) for p‐T231 + p‐S202/T205 (Figure 7A, **stacked bar plots**). These results demonstrate that the modification profiles of tau nano‐aggregates evolve with AD progression, with higher Braak stages showing an increased proportion of nano‐aggregates bearing mixed hyperphosphorylation signatures. This trend was similarly observed for intermediate‐aggregates, where there was a greater heterogeneity in the distribution of enrichment scores in AD (Braak 4) compared to AD (Braak 6) (Figure 7A, **violin plots**), and the percentage of dually modified intermediate‐aggregates increased in advanced AD (Figure 7A, **stacked bar plots**). Micro‐aggregates, on the other hand, were predominantly dually modified with both combinations of hyperphosphorylated residues visualized in both AD stages (Braak 4 and 6). These findings suggest that as tau aggregation advances, the aggregates accumulate more tau proteins that are modified at different residues, either through continued phosphorylation or via co‐aggregation of differentially modified tau seeds.

Given the changes in the phosphorylation patterns of tau nano‐aggregates across Braak stages, we sought to determine whether dually modified nano‐aggregates differed morphologically from their singly modified counterparts. ECLiPSE analysis revealed that dually modified nano‐aggregates exhibited reduced circularity, increased major axis length, and larger area compared to the singly modified nano‐aggregates (Figure 7B), resembling the morphological characteristics that distinguish nano‐aggregates in AD (Braak 4 and 6) from those observed in PART (Braak 1–3) and control cases.

Overall, these results reveal that the phosphorylation patterns of tau aggregates evolve as aggregation progresses, and that dually modified nano‐aggregates display distinct morphological features compared to their singly modified counterparts.

## 3 Conclusion

Utilizing DNA‐PAINT super‐resolution microscopy, we demonstrate the feasibility of detecting hyperphosphorylated nano‐sized tau aggregates (nano‐aggregates) within human postmortem brain tissues. Although the precise tau copy number within these nano‐aggregates remains unclear, their properties are consistent with tau oligomers, as evidenced by detection with the oligomer‐specific T22 antibody. However, the broad size range suggests a heterogeneous population encompassing a spectrum of tau assemblies, including insoluble protofibrils. Together, these findings suggest that nano‐aggregates represent early stages of tau aggregation. These results are further consistent with recent findings showing the presence of T22‐positive oligomeric tau in synapses within the cortex of AD brains.^[^ ^53^ ^]^ Given these observations, along with prior studies showing that the majority of tau aggregates are intraneuronal while extracellular aggregates, such as ghost tangles are rare,^[^ ^54^ ^]^ the tau nano‐aggregates detected in our DNA‐PAINT images are likely to be predominantly intraneuronal. Future studies incorporating neuronal compartment markers will be essential to further map the localization and enrichment of distinct tau aggregate species within the soma, neuronal processes, and synapses.

Importantly, our results demonstrate that p‐T181 and p‐T231 nano‐aggregates exist under physiological conditions, whereas p‐S202/T205 nano‐aggregates are specific to pathological cases, highlighting the sensitivity of DNA‐PAINT in detecting and quantifying the presence of both physiological and pathological tau nano‐aggregate species. A promising therapeutic strategy for tauopathies, such as AD, involves the use of tau‐targeting antibodies. Our findings suggest that antibodies directed against specific p‐tau residues, such as p‐S202/T205 may target pathological tau aggregates more selectively while sparing physiological ones, potentially preserving tau's normal function. Future work that uses a large cohort of age‐ and brain‐region‐matched samples is needed to further validate these findings. We focused on three specific p‐tau residues due to their significance in disease progression, their link to specific stages of tau aggregation, and the availability of highly specific and thoroughly validated antibodies.^[^ ^48^ ^]^ Looking ahead, it would be worthwhile to explore other post‐translational modifications of tau, including other p‐tau residues such as Threonine 217 (another tau biomarker), and ubiquitination marks, of which the latter has been shown biochemically to be enriched in tau oligomers.^[^ ^55^ ^]^ Such analysis will help clarify the differences in PTM profiles between physiological and pathological nano‐aggregates and provide insight into how these profiles evolve during disease progression.

In addition, leveraging the high resolution and quantitative nature of DNA‐PAINT, we demonstrated that nano‐aggregates exhibit distinct morphological characteristics in AD compared to controls. PART nano‐aggregates exhibited morphological features overlapping with both control and AD cases, suggesting that PART may lie on the disease continuum and represent a transitional state from healthy aging to AD. A limitation of our study is the small cohort size (N = 2 controls, N = 4 PART, N = 6 AD), reflecting the inherently low throughput of DNA‐PAINT imaging. However, recent advances in DNA‐PAINT acquisition speed^[^ ^56^ ^]^ will enable future studies in larger cohorts to more rigorously validate the transitional nature of PART pathology. Extending this approach to brain regions beyond the temporal and frontal cortex may also uncover region‐specific differences in tau nano‐aggregate profiles. Finally, applying this analysis to other tauopathies could determine whether each disorder is marked by morphologically distinct tau nano‐aggregates.

Multi‐color DNA‐PAINT analysis revealed that tau nano‐aggregates in advanced AD possess a unique hyperphosphorylation signature composed of a combination of p‐tau residues, such as both p‐T181 and p‐S202/T205 or p‐T231 and p‐S202/T205. This finding aligns with a recent study that employed single‐molecule pull‐down techniques to investigate the p‐tau composition of small tau aggregates extracted from human brain tissues and biofluids.^[^ ^57^ ^]^ Given the species overlap between the antibodies, we could not simultaneously examine more than two p‐tau residues. Nonetheless, it is probable that a subset of the nano‐aggregates carries all three phospho‐tau modifications. Here, we opted for secondary antibody detection due to its simplicity. In the future, directly conjugating primary antibodies with DNA‐PAINT compatible docking oligos will facilitate the simultaneous examination of a broader range of tau's post‐translational modifications to determine the combinatorial modification signature unique to different tau aggregates. Indeed, up to 30‐plex DNA‐PAINT imaging has been performed with a range of neuronal or cellular targets, highlighting the feasibility of this approach.^[^ ^58^, ^59^ ^]^

In contrast to advanced AD, nano‐aggregates from cases of intermediate AD and PART mainly contained only one of the p‐tau modifications visualized. This result is surprising, especially considering that p‐T181, one of the modifications we visualized, has previously been described as a “master” residue that, once phosphorylated, can lead to further phosphorylation at other residues.^[^ ^51^ ^]^ Our results suggest that the initial formation of nano‐aggregates likely does not require a combination of tau modifications, although we cannot exclude that other p‐tau modifications or PTMs may be enriched in these nano‐aggregates. However, as the disease progresses and aggregation continues, tau aggregates may undergo further hyperphosphorylation. It is also possible that nano‐aggregates with different modifications could coalesce, forming aggregates with combined modifications. Supporting this possibility, our analysis showed that nano‐aggregates with dual modifications were morphologically different from those with a single modification, often being larger and less circular. These combinatorially modified and morphologically distinct nano‐aggregates might exhibit greater toxicity and a higher propensity to seed further aggregation, particularly given their prevalence in advanced AD and the observation that micro‐aggregates are often dually modified. Future work comparing different tauopathies at various disease stages and utilizing markers for several disease‐relevant tau PTMs has the potential to reveal the specific modifications and morphological traits of tau seeds. This information may provide crucial insights into the critical modifications to target for inhibiting tau aggregation.

A limitation of studies relying on human postmortem brain tissue, including ours, is the possibility of postmortem dephosphorylation, which may lead to the underestimation of phospho‐tau levels. Super‐resolution imaging of freshly obtained brain biopsy material would represent a valuable future approach to address this limitation.

Together, our results uncover clear and progressive changes in both the hyperphosphorylation profiles and morphological features of tau nano‐aggregates across the spectrum, from healthy brains to PART and ultimately to AD. The improved capability to detect and quantitatively analyze tau nano‐aggregates in postmortem human brain tissue paves the way for new research into the molecular mechanisms of Alzheimer's Disease (AD) and related tauopathies.

## 4 Experimental Section

### Tissue Samples

Formalin‐fixed and paraffin embedded temporal cortex tissue blocks from three to four cases neuropathologically diagnosed with PART (Primary Age‐Related Tauopathy), Intermediate level of Alzheimer's disease, and high level of Alzheimer's disease neuropathologic change and control tissue (38‐yo and 60‐yo) were obtained from the University of Pennsylvania (Penn) Center for Neurodegenerative Disease Research (CNDR) Center as outlined in Table 1. Each tissue block was cut into 6 µm‐thick sections using a microtome and mounted over poly‐L‐Lysine‐coated coverslips. Mounted sections were incubated at 37 °C overnight and stored at room temperature.

### Tissue Preparation

Mounted tissue sections were incubated with xylene (99% vol/vol; Sigma Aldrich) two times (5 min per incubation). Samples were used right away after deparaffinization and were not stored. Deparaffinized samples were incubated with ethanol two times (1 min per incubation). Then samples were rehydrated by incubating them in a series of ethanol and deionized water solutions of 90%, 80%, and 70% (vol/vol; Thermo Fisher) (1 min per incubation). Lastly, samples were quickly rinsed with deionized water three times.

### Immunolabeling

The immunolabeling consisted of six main steps: antigen retrieval, reduction of autofluorescence, permeabilization, blocking, post‐fixation, and clearing.^[^ ^60^, ^61^ ^]^ For antigen retrieval, freshly deparaffinized and rehydrated samples were incubated with Tris‐EDTA buffer solution (10 mm Tris Base, 1 mm EDTA, 0.05% Tween‐20, PBS, pH 9.0) for 15 min at 94 °C and washed three times with Phosphate‐buffered saline (PBS) (5 min per wash). For reduction of autofluorescence, samples were incubated with freshly made 0.1% (wt/vol) sodium borohydride in PBS solution on ice for 15 min and washed three times with PBS (5 min per wash). Then, samples were permeabilized with 0.2% Triton x‐100 (vol/vol; Sigma‐Aldrich) in PBS for 30 min and washed three times with PBS (5 min per wash). Next, samples were blocked for 1 h with blocking solution (3% (wt/vol) bovine serum albumin, BSA, 0.2% Triton, PBS) at room temperature and washed three times with PBS (5 min per wash). They were then incubated overnight at 4 °C in a humidified chamber with the appropriate dilution of primary antibody in blocking solution and washed three times with PBS (5 min per wash). Samples were then incubated for 2 h at room temperature with a dilution of the appropriate secondary antibody and washed with PBS (5 min per wash). Then, samples were post‐fixed with 4% PFA in PBS for 10 min at room temperature and washed three times with PBS (5 min per wash). Lastly, samples were treated with 60% 2,2‐thiodiethanol (vol/vol; Sigma Aldrich) in PBS for 30 min at room temperature to reduce background and optical aberrations.

For the heat denaturation and antigen retrieval control sample (untreated), all the above steps were followed except for the incubation step with the antigen retrieval buffer at 94 °C. Sample was instead incubated with PBS at RT for 15 min, followed by the described steps for immunolabeling (reduction of autofluorescence, blocking, etc.).

For negative control samples, all the above steps were followed except for the addition of primary antibody.

Labeling of the samples for dual‐color imaging was carried out sequentially. Samples were first incubated with one of the primary antibodies using the protocol just described. After the incubation step with secondary antibody, samples were post‐fixed with 4% PFA in PBS for 10 min at room temperature and washed three times with PBS (5 min per wash). Samples were then blocked for 30 min with blocking solution (3% (wt/vol) BSA, 0.2% Triton, PBS) at room temperature and washed three times with PBS (5 min per wash). The samples were then incubated overnight at 4 °C in a humidified chamber with the appropriate dilution of the second primary antibody in blocking solution and washed three times with PBS (5 min per wash). Afterward, samples were incubated for 2 h at room temperature with a dilution of the appropriate secondary antibody and washed three times with PBS (5 min per wash). Then, samples were post‐fixed once again with 4% PFA in PBS for 10 min at room temperature and washed with PBS (5 min per wash). Lastly, samples were treated with 60% 2,2‐thiodiethanol (vol/vol; Sigma Aldrich) in PBS for 30 min at room temperature.

A list of primary and secondary antibodies used in these studies is provided in Tables 2 and **3**. The primary antibodies used for single‐color DNA‐PAINT acquisitions were: AT8 at a 1:50 dilution, AT180 at a 1:100 dilution, AT270 at a 1:50 dilution, and T22 at a 1:200 dilution. The secondary antibodies used in these studies were at a 1:100 dilution.

### Immunohistochemistry Labeling

Mounted paraffin‐embedded tissue sections were baked at 37 °C for 24–48 h before staining. The samples were deparaffinized in xylene two times (5 min per incubation) and rehydrated in 100% ethanol two times (1 min per incubation), followed by incubation in descending ethanol concentrations of 90%, 80%, and 70% (vol/vol; 1 min per incubation).

For heat‐induced antigen retrieval, samples were incubated in freshly prepared 5% hydrogen peroxide in methanol (vol/vol) for 30 min. The samples were then washed in running water for 15 min and microwaved at 90 °C for 15 min in Tris‐EDTA buffer (10 mm Tris‐base, 1 mm EDTA, 0.05% Tween‐20, pH 9.0). The samples remained in solution at room temperature for 30 min to enhance retrieval.

All samples were then washed in 0.1 m Tris buffer (pH 7.6) for 5 min and blocked in blocking buffer (0.1 m Tris/2% FBS (vol/vol)) for 5 min. The samples were incubated with primary antibody diluted in blocking buffer overnight (18–22 h) at 4 °C in a humidified chamber. They were then washed in 0.1 m Tris buffer for 5 min and then blocked in blocking buffer. Then, samples were incubated with a biotinylated secondary antibody in blocking buffer (1:500 dilution) for 90 min at room temperature. Samples were then washed in 0.1 m Tris buffer for 5 min and blocked again in blocking buffer for 5 min. VECTASTAIN Elite ABC‐HRP (Vector Laboratories) reagent was prepared by combining reagents A (avidin) and B (biotinylated HRP) with blocking buffer at a 1:500 dilution and incubating the solution at 4 °C for a minimum of 15 min before use. The samples were incubated with ABC‐HRP solution for 1 h at room temperature, followed by a wash in 0.1 m Tris buffer for 5 min. Next, samples were incubated for 10 min with ImmPACT DAB Peroxidase (HRP; Vector Laboratories) substrate to react with the avidin/biotinylated enzyme complex to generate a brown color, followed by three washes with Milli‐Q water (5 min per wash). The samples were counterstained with hematoxylin for 2 min and washed with Milli‐Q water for at least 1 min. They were then washed again with running water for 10 min and dehydrated through incubation in ascending ethanol concentrations (70%, 80%, 90%, and 100%; vol/vol; 1 min per incubation), followed by submersion in two xylene baths for 5 min each. Samples were then cover‐slipped for subsequent imaging and analysis.

### Immunofluorescence Labeling

The immunofluorescence labeling of samples consists of all the steps included in the “immunolabeling” section previously described, with the addition of a DAPI stain and the usage of different dilutions of primary and secondary antibodies (see Tables 2 and 3 in Experimental Section). After the tissue clearing step, samples were incubated with a (1:5000) DAPI solution (vol/vol) for 15 min, then washed three times with PBS (5 min per wash).

The primary antibodies (Table 2) used for single‐color IF acquisitions were: AT8 at a 1:200 dilution, AT180 at a 1:400 dilution, AT270 at a 1:200 dilution, and MAP2 at a 1:600 dilution. The secondary antibodies (Table 3) used for single‐color IF acquisitions were at a 1:100 dilution. AT8 labeled samples were imaged with Donkey anti‐mouse Alexa‐Fluor 546 (secondary antibody), and AT180, AT270, and MAP2 labeled samples were imaged with Donkey anti‐rabbit Alexa‐488 (secondary antibody).

### Dephosphorylating Control Assay

To dephosphorylate tissue sections, New England Biolab's Lambda Phosphatase protocol (Catalog number P0753S) and previous literature were followed.^[^ ^62^ ^]^ After sections were deparaffinized and the antigen retrieval step was performed, tissues were incubated with 200 µL of a solution containing Lambda Phosphatase in reaction buffer (20 000 units mL^−1^ phosphatase, 1x MnCl_2_, 1xNEBuffer, dH_2_O; New England Biolabs) at room temperature for 24 h. As a negative control, a mock treated sample was prepared, which consisted of a sample incubated with 200 µL of a solution containing 50% glycerol (vol/vol; Invitrogen) in reaction buffer (1xMnCl_2_, 1xNEBuffer, dH_2_O; New England Biolabs). After incubation, samples were washed three times with PBS (5 min per wash), followed by an incubation with Citrate Buffer (pH 6.0, Vector Laboratories) at 94 °C for 15 min. Samples were then thoroughly washed with PBS (5 min per wash). Treated and mock treated sample batches were then immunolabeled for IHC, IF, and DNA‐PAINT imaging following the respective protocols previously described. For DNA‐PAINT, mock and treated samples were imaged with imager probe 2‐Cy3b using the same DNA‐PAINT acquisition parameters as regular samples.

### Widefield Imaging

All widefield images were acquired using Zeiss Observer 7 inverted microscope with a Plan‐Apochromat 40x/0.95NA dry objective. All fields of view included in this manuscript were randomly selected and imaged. Additional image analysis, such as brightness and contrast adjustment and ROI selection, was performed using ImageJ/Fiji (NIH).^[^ ^63^ ^]^ Brightness and contrast were adjusted using consistent settings across all samples from each target for appropriate comparison. All IHC images were captured using the brightfield contrasting method with a TL LED (lightsource at 10%) and an exposure time of 15 ms.

### Confocal Imaging

All confocal images were acquired using a Zeiss LSM 880 with Airyscan and Fast Airyscan inverted confocal microscope, with a Plan‐Apochromat 20x/0.8NA dry objective. Z‐stacks were acquired in frame mode, at scan speed of 7 and using an average of 4. All fields of view included in this manuscript were randomly selected and imaged. Maximum intensity projections were generated using ImageJ/Fiji (NIH).^[^ ^63^ ^]^ Brightness and contrast were adjusted using consistent settings across all samples from each target for appropriate comparison. p‐S202/T205 IF images were acquired using 405 and 561 nm lasers at (0.500% and 1.8%) laser power, respectively. p‐T181 IF images were acquired using 405 and 488 nm lasers at (0.700% and 0.900%) laser power, respectively. p‐T231 IF images were acquired using 405 and 488 nm lasers at (0.600% and 0.600%) laser power, respectively.p‐T181 and p‐T231 IF images from mock dephosphorylated and dephosphorylated experiments were acquired using 405‐ and 488‐nm lasers at (4.0%, 0.600%) and (5.0%, 0.700%) laser powers, respectively. p‐S202/T205 IF images from the heat denaturation and antigen retrieval control experiment were acquired using 405 and 561 nm lasers at (0.5%, 2.8%) laser power.

Acquisition parameters for MAP2 IF images were individually optimized to capture MAP2 signal, while avoiding both oversaturation or underdetection. IF images were acquired using 405 and 488 nm lasers; 38‐yo sample was imaged at (1.8% and 3.0%) laser power, PART (Braak 2) sample was imaged at (1.8% and 1.2%) laser power, AD (Braak 4) sample was imaged at (1.2% and 0.800%) laser power, and AD (Braak 6) was imaged at (1.8% and 3.0%) laser power, respectively.

### DNA‐PAINT Imaging

All DNA‐PAINT images were acquired using the Oxford Nanoimaging System. All fields of view included in this manuscript were randomly selected and imaged. The Nanoimager‐S microscope has the following configuration: 405, 488, 561, and 640 nm lasers, 498–551 and 576–620 nm band‐pass filters in channel 1, and 666–705 nm band‐pass filters in channel 2, 100x 1.45 NA oil immersion objective (Olympus), and Hamamatsu Flash 4 V3 sCMOS camera. All single‐and dual‐color acquisitions were conducted at 27 °C using HiLo illumination and illumination with 10 mW 647 nm laser and 2.5 mW 561 nm laser with an exposure time of 100 ms.

For single‐color DNA‐PAINT acquisitions, samples and negative controls were incubated with a solution of appropriate imager‐oligo in imaging buffer (0.5 nm; vol/vol). AT8 labeled samples were imaged with imager oligo 1‐ATTO655, and AT180, AT270, and T22 labeled samples were imaged with imager oligo 2‐Cy3b. Negative control samples labeled with anti‐mouse docking site 1 (secondary antibody) were imaged with imager oligo 1‐ATTO655, whereas negative samples labeled with anti‐rabbit docking site 2 (secondary antibody) were imaged with imager oligo 2‐Cy3b. All images were acquired at a 100 ms exposure for 34000 frames. The imager‐ oligo solution was changed every 4 images (every ≈4 h). Before the addition of a fresh imager‐oligo solution, samples were thoroughly washed 5 times with PBS (3 min per wash).

For dual‐color DNA‐PAINT acquisitions, samples were incubated with a solution of appropriate imager‐oligos (0.5 nM; vol/vol) in imaging buffer. AT8, AT180 dually labeled samples were imaged with a solution of imager oligos 1 and 2 (ATTO655, Cy3b), and AT8, AT270 dually labeled samples were imaged with a solution of imager oligos 1 and 2 (ATTO655, Cy3b). Dual‐color images were acquired at a 100 ms exposure using a laser program in which the 561‐ and 647‐ lasers were sequentially activated every 200 frames, capturing a total of 68000 frames, 34000 frames per target. The solution of imager‐probes was changed every 2 images (every ≈4 h). Before the addition of a fresh imager‐probe solution, samples were thoroughly washed five times with PBS (3 min per wash).

### Single‐Color Confocal‐IF Images Voronoi Tessellation and Segmentation

The images from the heat denaturation and antigen retrieval control experiment (treated and untreated samples) were analyzed using ImageJ/Fiji(NIH).^[^ ^63^ ^]^ To identify tau aggregates, the threshold values were adjusted utilizing the images from the treated sample. These values were then used consistently across all images from the untreated sample for appropriate comparison. Afterward, a mask was generated and analyzed using the “analyze particles” function. To reduce background and noise, the area threshold was set to (2‐infinity) µm^2^. It was visually confirmed that the established threshold was removing potential labeling artifacts and not tau aggregates. Subsequent data analysis and plotting were performed using MATLAB R2022a and GraphPad Prism.

### Single‐Color DNA‐PAINT Images

For single‐color acquisitions, localizations were exported in.csv files using the NimOS localization software and rendered using a custom‐written data analysis software (https://github.com/melikelakadamyali/StormAnalysisSoftware; MATLAB R2022a). All subsequent data analysis was performed using this custom‐written software. For data segmentation, Voronoi tessellation was first performed, in which each localization in space is associated with a Voronoi polygon based on its neighboring localizations.^[^ ^49^ ^]^ The Voronoi polygon area is a measure of localization density, with dense localizations having small Voronoi polygons areas and vice versa. The Voronoi polygon area can therefore be used as a threshold to cluster together localizations in dense regions for segmentation.^[^ ^49^ ^]^ The area threshold for images from each case was adjusted accordingly to account for differences in tau aggregate burden and localization density: PART (Braak 1–3) = (0.00018–0.0003 µm^2^); AD (Braak 4) = (0.001–0.002 µm^2^); AD (Braak 6) = (>0.002 µm^2^). Additionally, a threshold of a minimum of 10 localizations was employed, in which segmented objects having less than 10 localizations were discarded. It was visually confirmed that the selected thresholds properly segmented individual tau aggregates in the single‐color super‐resolution images. These thresholds were applied consistently across all imaged brain tissues.

### Removal of Imaging Artefacts

Segmented objects have a positive correlation between the area value and the number of localizations. Objects that deviate from this trend (i.e., that have a small area value and a large number of localizations, or large area values and a small number of localizations) were considered to correspond to imaging artefacts.^[^ ^44^ ^]^ These outlier objects were further filtered out from the lists of segmented tau aggregates for all images.

### Removal of Background and Noise

Images of negative control samples were segmented using the same Voronoi polygon area threshold and minimum number of localizations as described above. The area distribution of the resulting segmented objects was plotted and compared to the area distribution of objects from the corresponding positive control samples. An area threshold was then imposed based on this comparison to remove additional background and noise. For samples imaged with the imager‐oligo 2‐Cy3b, the area threshold was 0.004 µm^2^, and for those imaged with imager oligo 1‐ATTO655, the area threshold was 0.006 µm^2^. The area thresholds were slightly different for the two imager‐oligos as they gave rise to slightly different levels of background/noise. Applying these thresholds to the negative control samples removed >97% of segmented objects corresponding to background and noise. Hence, these thresholds were used for removing background and noise present in the positive control samples imaged using the corresponding imager‐oligos.

### Area‐Based Classification of Segmented Tau Aggregates

To separate segmented tau aggregates into distinct classes, the area distribution of tau aggregates across cases and establish specific area thresholds for separating segmented tau aggregates into three distinct area‐based classes (Class I: nano‐aggregates, Class II: intermediate‐sized aggregates, Class III: micro‐sized aggregates) were evaluated. The area thresholds used were 0.004–0.017 µm^2^ (for aggregates visualized using imager probe 2‐Cy3b)) or 0.006–0.017 µm^2^ (for aggregates visualized using imager probe 1‐ATTO655) for Class I, 0.017–0.15 µm^2^ for Class II, and >0.15 µm^2^ for Class III. Note that the minimum area threshold for Class I was slightly different for the nano‐aggregates imaged using the two different imager‐probes as described above. These thresholds were applied consistently across all images. It was visually confirmed that tau aggregates from single‐color acquisitions from all cases were properly classified.

### ECLiPSE Analysis

ECLiPSE is an automated shape quantification and classification method that is capable of determining morphological properties of structures imaged using super‐resolution microscopy.^[^ ^50^ ^]^ For detailed information on the ECLiPSE methodology, the reader is referred.^[^ ^50^ ^]^ Briefly, the localized point clouds corresponding to individual clusters were used, without any additional rendering, to calculate 67 different morphological and structural descriptors (i.e., geometric, boundary, skeleton, fractal, etc. descriptors). The use of the unrendered point cloud data provides the most unbiased quantification of the morphological properties.

Once all properties were calculated with ECLiPSE, Principal Component Analysis (PCA) was used to capture the essential information in the data (after unit variance scaling to remove scaling artifacts). PCA is a compression technique that transforms data into latent variables (called principal components, PCs) by using a linear combination of the original variables to maximize data variability. These latent variables are uncorrelated with respect to each other and will create a new coordinate system onto which the original data can be projected. As each latent variable explains more data variability than the subsequent one, the first few PCs retain most of the variability of the original data set. This allows us to simplify complex data sets while retaining the essential characteristics of the data (i.e., dimensionality reduction). This property of PCA also explains that data points that are spatially close in the PCA space will have similar features (i.e., biological properties obtained using ECLiPSE), and therefore, two sets of data points that overlap will have similar biological properties, whereas two sets of data points that do not overlap will have different biological properties. The overlap of the two sets was determined using the polyshape (MATLAB) representation, and then the Jaccard index was calculated between the two (i.e., the ratio between the intersection of the two sets divided by the sum of the two sets). After constructing the PCA space and projecting the data points into this new coordinate system, each data point was color‐coded according to which case it belongs to for visual purposes, PART (Braak 1‐3), AD (Braak 4), or AD (Braak 6).

### Statistical Analysis

All unpaired two‐sided and one‐sided rank sum tests performed in these studies were calculated using MATLAB, version R2022a. An unpaired one‐sided rank sum test was performed in Figure 2D to test the hypothesis that dephosphorylation leads to a decrease in the number of phospho‐tau nano‐aggregates, whereas an unpaired two‐sided rank sum test was performed in the rest of the manuscript. A *p* value <0.05 was taken as statistically significant. *P* values: ns (>0.05), * (0.05–0.01), ** (0.001–0.01), *** (0.0001–0.001), **** (< 0.0001).

### Dual‐Color DNA‐PAINT Images Voronoi Tessellation and Segmentation

For dual‐color acquisitions, a strategy similar to that of single‐color acquisitions was applied, with the exception that before Voronoi tessellation, channels pertaining to the localizations from each target were combined to generate a merged reference image. Segmentation was then performed as described above followed by the removal of imaging artifacts and classification of segmented tau aggregates into area‐based classes.

To correct segmentation errors originating from the merging of channels, the segmentation quality was evaluated by calculating the overlap score of the localizations belonging to the two distinct channels within an individual aggregate. The overlap score was computed by first transforming the localizations of each channel into an alpha‐shape (i.e., a special case of Voronoi polygons that is a precise description of the aggregates) and then determining the intersection between these two alpha‐shape objects. The overlap score is then calculated as the percentage of area that overlaps (i.e., the area of this intersection) with the alpha‐shape object of the localizations in the other channel. This was evaluated with respect to both channels, and the overlap scores were consistent, regardless of which channel was used as a reference. Segmented aggregates were considered with an overlap score <30% to be two distinct aggregates that were spatially proximate and became merged in the reference image in error. To rectify such segmentation errors, an area filter (above 0.15 µm^2^) was first applied to remove all micron‐sized aggregates as these all had an overlap score greater than 30% and did not need to be corrected. Aggregates below this area threshold with an overlap score of <30% were re‐segmented. These now distinct aggregates were also reassigned to the correct area‐based class (i.e., nano‐, intermediate‐, or micro‐aggregate) if needed. Resulting lists of segmented aggregates were combined and visually inspected for proper segmentation once again. These thresholds and re‐segmentation parameters were applied consistently across different conditions.

### Colocalization Analysis

Additionally, to evaluate the composition of the different aggregates, an enrichment score was calculated. This enrichment score is obtained by calculating the ratio between the difference in number of localizations in each channel of the aggregate and the total number of localizations for the aggregate. An enrichment score of −1 or 1 means that the aggregate completely consists of a single type of phospho‐tau marker, whereas an enrichment score of 0 represents an equal distribution of phospho‐tau markers for that aggregate. Anything between −1 and 0, or 0 and 1 will contain more of one phospho‐tau marker than the other.

### Ethics Approval and Patient Consent Statement

Informed consent was obtained from next of kin for all autopsies in accordance with local laws and regulations. Furthermore, autopsy studies are not legally considered human subjects research, and so oversight from the University of Pennsylvania Institutional Review Board has been deferred.

## Conflict of Interest

The authors declare no conflict of interest.

## Author Contributions

M.L. and A.N.S.R. contributed to the conceptualization. A.N.S.R., S.H., G. L.C., and C.R.B. contributed to the methodology. A.N.S.R. and S.H. carried out the investigation and analysis. A.N.S.R performed visualization. E.B.L. and G.L.C. arranged materials. M.L. and E.B.L. supervised the project. M.L. and A.N.S.R. wrote the original draft. M.L., A.N.S.R., S.H., G.L.C., C.R.B., and E.B.L. wrote, reviewed and edited the manuscript.

## Supporting information



Supporting Information

## Data Availability

The processed and analyzed data in this manuscript has been deposited to Figshare (https://doi.org/10.6084/m9.figshare.25673916.v1). The code has been deposited to https://github.com/melikelakadamyali/StormAnalysisSoftware. ECLIPSE software has been deposited to https://github.com/LakGroup/ECLiPSE. The raw imaging data is too large to deposit to a public repository and is available from authors upon request. All other data are available in the main text and supplementary materials.

## References

[1] T. Guo , W. Noble , D. P. Hanger , Acta Neuropathol. 2017, 133, 665. 28386764 10.1007/s00401-017-1707-9 PMC5390006

[2] V. M. Lee , M. Goedert , J. Q. Trojanowski , Annu. Rev. Neurosci. 2001, 24, 1121. 11520930 10.1146/annurev.neuro.24.1.1121

[3] Y. Wang , E. Mandelkow , Nat. Rev. Neurosci. 2016, 17, 22. 26631930 10.1038/nrn.2015.1

[4] A. Cario , C. L. T. Berger , BioEssays 2023, 45, 2200138. 10.1002/bies.202200138 PMC10630968 37489532

[5] L. Balabanian , D. V. Lessard , K. Swaminathan , P. Yaninska , M. Sébastien , S. Wang , P. W. Stevens , P. W. Wiseman , C. L. Berger , A. G. Hendricks , Mol. Biol. Cell 2022, 33, ar128. 36129768 10.1091/mbc.E22-01-0018 PMC9634973

[6] J. L. Stern , D. V. Lessard , G. J. Hoeprich , G. A. Morfini , C. L. Berger , Mol. Biol. Cell 2017, 28, 1079. 28251926 10.1091/mbc.E16-10-0728 PMC5391184

[7] R. Dixit , J. L. Ross , Y. E. Goldman , E. L. Holzbaur , Science 2008, 319, 1086. 18202255 10.1126/science.1152993 PMC2866193

[8] N. Samudra , C. Lane‐Donovan , L. VandeVrede , A. L. Boxer , J. Clin. Invest. 2023, 133, 168553. 10.1172/JCI168553 PMC10266783 37317972

[9] Y. Zhang , K.‐M. Wu , L. Yang , Q. Dong , J.‐T. Yu , Mol. Neurodegenerat. 2022, 17, 28. 10.1186/s13024-022-00533-z PMC8991707 35392986

[10] J. Gotz , G. Halliday , R. M. Nisbet , Annu Rev Pathol 2019, 14, 239. 30355155 10.1146/annurev-pathmechdis-012418-012936

[11] M. G. Spillantini , M. Goedert , Lancet Neurol. 2013, 12, 609. 23684085 10.1016/S1474-4422(13)70090-5

[12] L. I. Binder , A. L. Guillozet‐Bongaarts , F. Garcia‐Sierra , R. W. T. Berry , Biochim. Biophys. Acta 2005, 1739, 216. 15615640 10.1016/j.bbadis.2004.08.014

[13] H. Braak , E. Braak , Acta Neuropathol. 1991, 82, 239. 1759558 10.1007/BF00308809

[14] T. J. Montine , C. H. Phelps , T. G. Beach , E. H. Bigio , N. J. Cairns , D. W. Dickson , C. Duyckaerts , M. P. Frosch , E. Masliah , S. S. Mirra , P. T. Nelson , J. A. Schneider , D. R. Thal , J. Q. Trojanowski , H. V. Vinters , B. T. Hyman , Acta Neuropathol. 2012, 123, 1. 22101365 10.1007/s00401-011-0910-3 PMC3268003

[15] J. A. Trejo‐Lopez , A. T. Yachnis , S. Prokop , Neurotherapeutics 2022, 19, 173. 34729690 10.1007/s13311-021-01146-y PMC9130398

[16] T. Vogels , A. Leuzy , C. Cicognola , N. J. Ashton , T. Smolek , M. Novak , K. Blennow , H. Zetterberg , T. Hromadka , N. Zilka , M. Schöll , Biol. Psychiatry 2020, 87, 808. 31735253 10.1016/j.biopsych.2019.09.019

[17] H. Braak , I. Alafuzoff , T. Arzberger , H. Kretzschmar , K. Del Tredici , Acta Neuropathol. 2006, 112, 389. 16906426 10.1007/s00401-006-0127-z PMC3906709

[18] N. Franzmeier , J. Neitzel , A. Rubinski , R. Smith , O. Strandberg , R. Ossenkoppele , O. Hansson , M. Ewers , M. Weiner , P. Aisen , R. Petersen , C. R. Jack , W. Jagust , J. Q. Trojanowki , A. W. Toga , L. Beckett , R. C. Green , A. J. Saykin , J. Morris , L. M. Shaw , E. Liu , T. Montine , R. G. Thomas , M. Donohue , S. Walter , D. Gessert , T. Sather , G. Jiminez , D. Harvey , M. Donohue , et al., Nat. Commun. 2020, 11, 347.31953405

[19] L. Martin , X. Latypova , F. Terro , Neurochem. Int. 2011, 58, 458. 21215781 10.1016/j.neuint.2010.12.023

[20] C. Alquezar , S. Arya , A. W. Kao , Front Neurol 2020, 11, 595532. 33488497 10.3389/fneur.2020.595532 PMC7817643

[21] G. Simic , M. Babic Leko , S. Wray , C. Harrington , I. Delalle , N. Jovanov‐Milosevic , D. Bazadona , L. Buée , R. De Silva , G. Di Giovanni , C. Wischik , P. Hof , Biomolecules 2016, 6, 6. 26751493 10.3390/biom6010006 PMC4808800

[22] C. X. Gong , K. Iqbal , Curr. Med. Chem. 2008, 15, 2321. 18855662 10.2174/092986708785909111 PMC2656563

[23] E. Köpke , Y. C. Tung , S. Shaikh , A. C. Alonso , K. Iqbal , I. Grundke‐Iqbal , J. Biol. Chem. 1993, 268, 24374. 8226987

[24] F. Liu , B. Li , E.‐J. Tung , I. Grundke‐Iqbal , K. Iqbal , C.‐X. Gong , Eur J Neurosci 2007, 26, 3429. 18052981 10.1111/j.1460-9568.2007.05955.x PMC2262108

[25] E. Ercan , S. Eid , C. Weber , A. Kowalski , M. Bichmann , A. Behrendt , F. Matthes , S. Krauss , P. Reinhardt , S. Fulle , D. E. Ehrnhoefer , Molecular Neurodegeneration 2017, 12, 87. 29157277 10.1186/s13024-017-0229-1 PMC5697095

[26] M. Goedert , R. Jakes , E. Vanmechelen , Neurosci. Lett. 1995, 189, 167. 7624036 10.1016/0304-3940(95)11484-e

[27] J. Neddens , M. Temmel , S. Flunkert , B. Kerschbaumer , C. Hoeller , T. Loeffler , V. Niederkofler , G. Daum , J. Attems , B. Hutter‐Paier , Acta Neuropathologica Communications 2018, 6, 52. 29958544 10.1186/s40478-018-0557-6 PMC6027763

[28] L. Amniai , G. Lippens , I. Landrieu , Biochem. Biophys. Res. Commun. 2011, 412, 743. 21871442 10.1016/j.bbrc.2011.08.046

[29] G. A. Jicha , E. Lane , I. Vincent , L. Otvos , R. Hoffmann , P. Davies , J. Neurochem. 1997, 69, 2087. 9349554 10.1046/j.1471-4159.1997.69052087.x

[30] J. C. Augustinack , A. Schneider , E. M. Mandelkow , B. T. Hyman , Acta Neuropathol. 2002, 103, 26. 11837744 10.1007/s004010100423

[31] M. Suárez‐Calvet , T. K. Karikari , N. J. Ashton , J. Lantero Rodríguez , M. Milà‐Alomà , J. D. Gispert , G. Salvadó , C. Minguillon , K. Fauria , M. Shekari , O. Grau‐Rivera , E. M. Arenaza‐Urquijo , A. Sala‐Vila , G. Sánchez‐Benavides , J. M. González‐de‐Echávarri , G. Kollmorgen , E. Stoops , E. Vanmechelen , H. Zetterberg , K. Blennow , J. L. Molinuevo , A. Beteta , R. Cacciaglia , A. Cañas , C. Deulofeu , I. Cumplido , R. Dominguez , M. Emilio , C. Falcon , S. Fuentes , et al., EMBO Mol. Med. 2020, 12, 12921.

[32] C. Schaffer , N. Sarad , A. DeCrumpe , D. Goswami , S. Herrmann , J. Morales , P. Patel , J. Osborne , J Lab Autom 2015, 20, 589. 25424384 10.1177/2211068214559979

[33] Y. Carlomagno , S. Manne , M. DeTure , M. Prudencio , Y.‐J. Zhang , R. Hanna Al‐Shaikh , J. A. Dunmore , L. M. Daughrity , Y. Song , M. Castanedes‐Casey , L. J. Lewis‐Tuffin , K. A. Nicholson , Z. K. Wszolek , D. W. Dickson , A. W. Fitzpatrick , L. Petrucelli , C. N. Cook , Cell Rep. 2021, 34, 108843. 33730588 10.1016/j.celrep.2021.108843 PMC8094113

[34] M. Usenovic , S. Niroomand , R. E. Drolet , L. Yao , R. C. Gaspar , N. G. Hatcher , J. Schachter , J. J. Renger , S. Parmentier‐Batteur , J. Neurosci. 2015, 35, 14234. 26490863 10.1523/JNEUROSCI.1523-15.2015 PMC6605424

[35] C. Kim , T. Haldiman , S.‐G. Kang , L. Hromadkova , Z. Z. Han , W. Chen , F. Lissemore , A. Lerner , R. de Silva , M. L. Cohen , D. Westaway , J. G. Safar , Sci. Transl. Med. 2022, 14, abg0253. 10.1126/scitranslmed.abg0253 PMC9549419 34985969

[36] C. A. Lasagna‐Reeves , D. L. Castillo‐Carranza , U. Sengupta , J. Sarmiento , J. Troncoso , G. R. Jackson , R. Kayed , FASEB J. 2012, 26, 1946. 22253473 10.1096/fj.11-199851 PMC4046102

[37] A. Van der Jeugd , K. Hochgräfe , T. Ahmed , J. M. Decker , A. Sydow , A. Hofmann , D. Wu , L. Messing , D. Balschun , R. D'Hooge , E.‐M. Mandelkow , Acta Neuropathol. 2012, 123, 787. 22532069 10.1007/s00401-012-0987-3 PMC4979687

[38] C. A. Lasagna‐Reeves , D. L. Castillo‐Carranza , U. Sengupta , A. L. Clos , G. R. Jackson , R. Kayed , Mol. Neurodegenerat. 2011, 6, 39. 10.1186/1750-1326-6-39 PMC3224595 21645391

[39] D. L. Castillo‐Carranza , J. E. Gerson , U. Sengupta , M. J. Guerrero‐Muñoz , C. A. Lasagna‐Reeves , R. Kayed , J Alzheimers Dis 2014, 40 Suppl 1, S97. 24603946 10.3233/JAD-132477

[40] S. S. Shafiei , M. J. Guerrero‐Munoz , D. L. Castillo‐Carranza , Front Aging Neurosci 2017, 9, 83. 28420982 10.3389/fnagi.2017.00083 PMC5378766

[41] X. Q. Chen , W. C. Mobley , Front Neurosci 2019, 13, 659. 31293377 10.3389/fnins.2019.00659 PMC6598402

[42] J. E. Gerson , D. L. Castillo‐Carranza , R. Kayed , ACS Chem. Neurosci. 2014, 5, 752. 25075869 10.1021/cn500143n

[43] C. Bond , A. N. Santiago‐Ruiz , Q. Tang , M. Lakadamyali , Mol. Cell 2022, 82, 315. 35063099 10.1016/j.molcel.2021.12.022 PMC8852216

[44] M. T. Gyparaki , A. Arab , E. M. Sorokina , A. N. Santiago‐Ruiz , C. H. Bohrer , J. Xiao , M. Lakadamyali , Proc Natl Acad Sci U S A 2021, 118. 10.1073/pnas.2021461118 PMC8126857 33952699

[45] J. L. Guo , A. Buist , A. Soares , K. Callaerts , S. Calafate , F. Stevenaert , J. P. Daniels , B. E. Zoll , A. Crowe , K. R. Brunden , D. Moechars , V. M. Y. Lee , J. Biol. Chem. 2016, 291, 13175. 27129267 10.1074/jbc.M115.712083 PMC4933232

[46] R. Jungmann , M. S. Avendaño , J. B. Woehrstein , M. Dai , W. M. Shih , P. Yin , Nat. Methods 2014, 11, 313. 24487583 10.1038/nmeth.2835 PMC4153392

[47] J. F. Crary , J. Q. Trojanowski , J. A. Schneider , J. F. Abisambra , E. L. Abner , I. Alafuzoff , S. E. Arnold , J. Attems , T. G. Beach , E. H. Bigio , N. J. Cairns , D. W. Dickson , M. Gearing , L. T. Grinberg , P. R. Hof , B. T. Hyman , K. Jellinger , G. A. Jicha , G. G. Kovacs , D. S. Knopman , J. Kofler , W. A. Kukull , I. R. Mackenzie , E. Masliah , A. McKee , T. J. Montine , M. E. Murray , J. H. Neltner , I. Santa‐Maria , W. W. Seeley , et al., Acta Neuropathol. 2014, 128, 755. 25348064 10.1007/s00401-014-1349-0 PMC4257842

[48] M. J. Ellis , C. Lekka , H. Tulmin , F. Seedat , D. P. O'Brien , S. Dhayal , M.‐L. Zeissler , J. G. Knudsen , B. M. Kessler , N. G. Morgan , J. A. Todd , S. J. Richardson , M. I. Stefana , Acta Neuropathol. 2024, 147, 87. 38761203 10.1007/s00401-024-02729-7 PMC11102361

[49] F. Levet , E. Hosy , A. Kechkar , C. Butler , A. Beghin , D. Choquet , J.‐B. Sibarita , Nat. Methods 2015, 12, 1065. 26344046 10.1038/nmeth.3579

[50] S. Hugelier , Q. Tang , H. Hyun‐Sook Kim , M. T. Gyparaki , C. Bond , A. N. Santiago‐Ruiz , S. Porta , M. Lakadamyali , Nat Methods. 2024, 21, 1909, 10.1038/s41592-024-02414-3. 39256629 PMC11466814

[51] K. Stefanoska , M. Gajwani , A. R. P. Tan , H. I. Ahel , P. R. Asih , A. Volkerling , A. Poljak , A. Ittner , Sci. Adv. 2022, 8, abl8809. 10.1126/sciadv.abl8809 PMC9258953 35857446

[52] R. Jungmann , M. S. Avendaño , M. Dai , J. B. Woehrstein , S. S. Agasti , Z. Feiger , A. Rodal , P. Yin , Nat. Methods 2016, 13, 439. 27018580 10.1038/nmeth.3804 PMC4941813

[53] M. Colom‐Cadena , C. Davies , S. Sirisi , J.‐E. Lee , E. M. Simzer , M. Tzioras , M. Querol‐Vilaseca , É. Sánchez‐Aced , Y. Y. Chang , K. Holt , R. I. McGeachan , J. Rose , J. Tulloch , L. Wilkins , C. Smith , T. Andrian , O. Belbin , S. Pujals , M. H. Horrocks , A. Lleó , T. L. Spires‐Jones , Neuron 2023, 111, 2176. 10.1016/j.neuron.2023.04.020 37192625

[54] T. J. Zwang , B. Woost , J. Bailey , Z. Hoglund , D. S. Richardson , R. E. Bennett , B. T. Hyman , Brain Commun 2023, 5, fcad130. 37324243 10.1093/braincomms/fcad130 PMC10263274

[55] N. Puangmalai , U. Sengupta , N. Bhatt , S. Gaikwad , M. Montalbano , A. Bhuyan , S. Garcia , S. McAllen , M. Sonawane , C. Jerez , Y. Zhao , R. Kayed , J. Biol. Chem. 2022, 298, 101766. 35202653 10.1016/j.jbc.2022.101766 PMC8942844

[56] S. Strauss , R. Jungmann , Nat. Methods 2020, 17, 789. 32601424 10.1038/s41592-020-0869-x PMC7610413

[57] D. Boken , D. Cox , M. Burke , J. Y. L. Lam , T. Katsinelos , J. S. H. Danial , E. Fertan , W. A. McEwan , J. B. Rowe , D. Klenerman , Angew Chem Int Ed Engl 2024, 63, 202317756. 10.1002/anie.202317756 PMC11497306 38523073

[58] E. M. Unterauer , S. Shetab Boushehri , K. Jevdokimenko , L. A. Masullo , M. Ganji , S. Sograte‐Idrissi , R. Kowalewski , S. Strauss , S. C. M. Reinhardt , A. Perovic , C. Marr , F. Opazo , E. F. Fornasiero , R. Jungmann , Cell 2024, 187, 1785. 38552614 10.1016/j.cell.2024.02.045

[59] F. Schueder , F. Rivera‐Molina , M. Su , Z. Marin , P. Kidd , J. E. Rothman , D. Toomre , J. Bewersdorf , Cell 2024, 187, 1769. . 38552613 10.1016/j.cell.2024.02.033 PMC12135969

[60] J. Xu , H. Ma , H. Ma , W. Jiang , C. A. Mela , M. Duan , S. Zhao , C. Gao , E.‐R. Hahm , S. M. Lardo , K. Troy , M. Sun , R. Pai , D. B. Stolz , L. Zhang , S. Singh , R. E. Brand , D. J. Hartman , J. Hu , S. J. Hainer , Y. Liu , Nat. Commun. 2020, 11, 1899. 32313005 10.1038/s41467-020-15718-7 PMC7171144

[61] C. L. German , M. V. Gudheti , A. E. Fleckenstein , E. M. Jorgensen , Super‐Resolution Microscopy: Methods and Protocols, (Ed: H. Erfle ), Springer, New York, 2017, pp. 153‐162. 10.1007/978-1-4939-7265-4_13 PMC5814113 28924666

[62] M. J. Ellis , C. Lekka , K. L. Holden , H. Tulmin , F. Seedat , D. P. O'Brien , S. Dhayal , M.‐L. Zeissler , J. G. Knudsen , B. M. Kessler , N. G. Morgan , J. A. Todd , S. J. Richardson , M. I. Stefana , Acta Neuropathol. 2024, 147, 87. 38761203 10.1007/s00401-024-02729-7 PMC11102361

[63] J. Schindelin , I. Arganda‐Carreras , E. Frise , V. Kaynig , M. Longair , T. Pietzsch , S. Preibisch , C. Rueden , S. Saalfeld , B. Schmid , J.‐Y. Tinevez , D. J. White , V. Hartenstein , K. Eliceiri , P. Tomancak , A. Cardona , Nat. Methods 2012, 9, 676. 22743772 10.1038/nmeth.2019 PMC3855844

