# Supplementary material for: Morphological and Hyperphosphorylation Transitions of Nanoscale Tau Aggregates in Alzheimer's Disease
Source: Adv Sci (Weinh). 2025 Nov 19;13(6):e09773. doi: 10.1002/advs.202509773 (PMC12866830; doi:10.1002/advs.202509773)
Supplement: Supplementary file 1 — Supporting Information [file ADVS-13-e09773-s001.docx]

Supporting Information

**Morphological and Hyperphosphorylation Transitions of Nanoscale Tau Aggregates in Alzheimer’s Disease**

*Adriana N. Santiago-Ruiz, Siewert Hugelier, Gabriela L. Correa, Charles R. Bond, Edward B. Lee, Melike Lakadamyali**


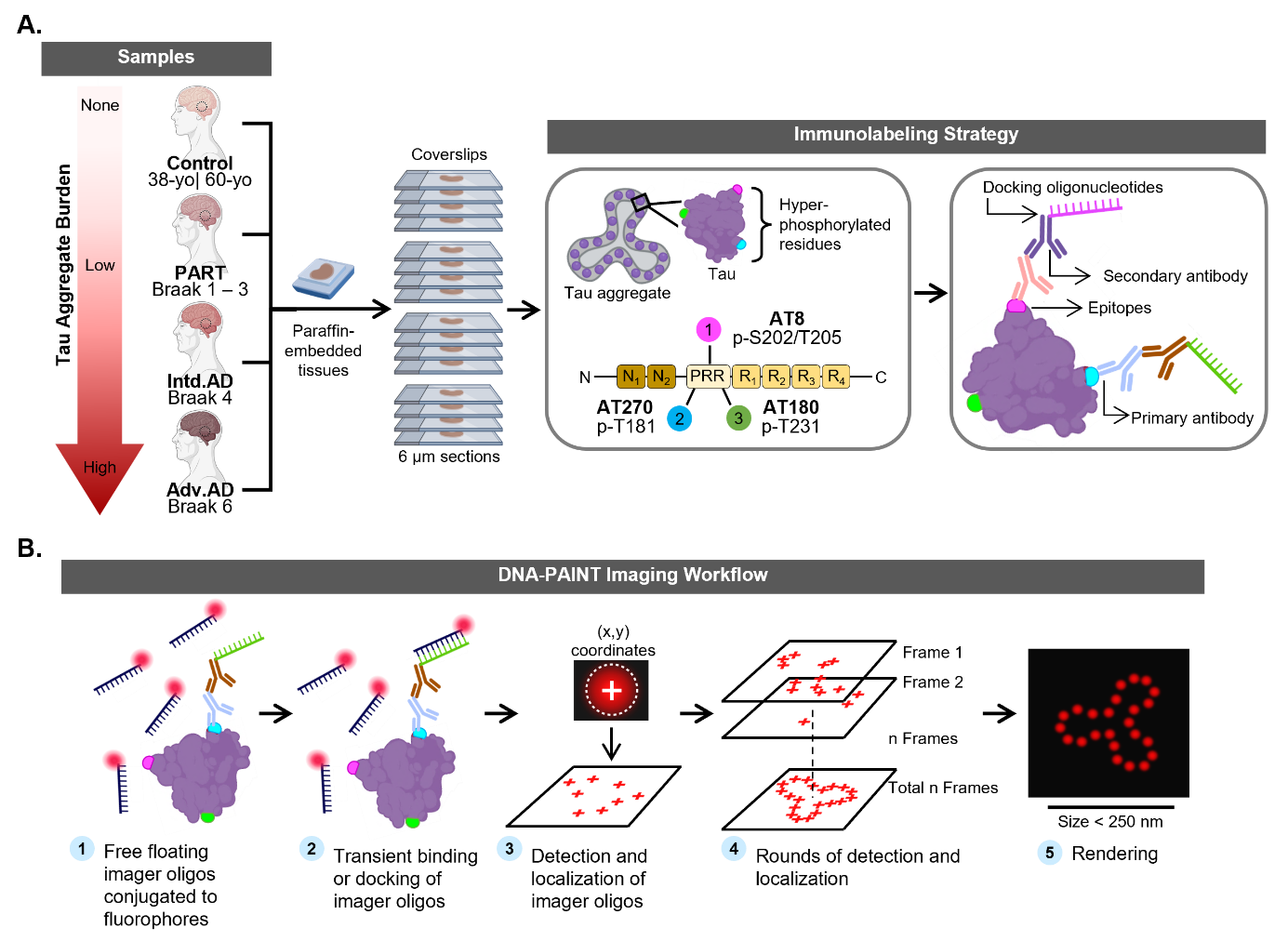


**Figure S1.** Schematic representation of sample preparation and imaging. A) Tissue blocks from a total of 10 patients neuropathologically diagnosed with PART (N=4; Braak 1-3), Intermediate (N=3; Braak 4) and Advanced (N=3; Braak 6) AD, and control cases lacking any tau pathology (N=2) were sectioned into 6 μm thick sections and mounted onto treated coverslips. A minimum of 16 sections per patient were obtained. Sections were immunolabeled with validated primary antibodies to target disease-confirmed hyperphosphorylated tau residues, Serine 202/Threonine 205 (p-S202/T205), Threonine 181 (p-T181), and Threonine 231 (p-T231). All sites are located in Tau’s Proline-Rich Region (PRR). After incubation with the primary antibody, a secondary antibody conjugated to a unique DNA-sequence (docking oligonucleotide) is added. B) Super-resolution images are acquired by performing DNA-PAINT. The schematic diagram shows a summary of the imaging process: 1) immunolabeled samples are incubated with a solution of imager oligos, which constitute fluorophore (ATTO655 or Cy3b) conjugated DNA-sequences complementary to the docking oligos; 2) these free floating imager oligos transiently bind to the docking oligo, allowing for the detection of a signal; 3) this information is then utilized to calculate the relative position of that fluorophore in space (x, y position, i.e. localizations); 4) the cycle is then repeated for a number of frames to accumulate a collection of localizations; 5) at the end of the acquisition, all these frames are merged to render a high-resolution image of the labeled object of interest.


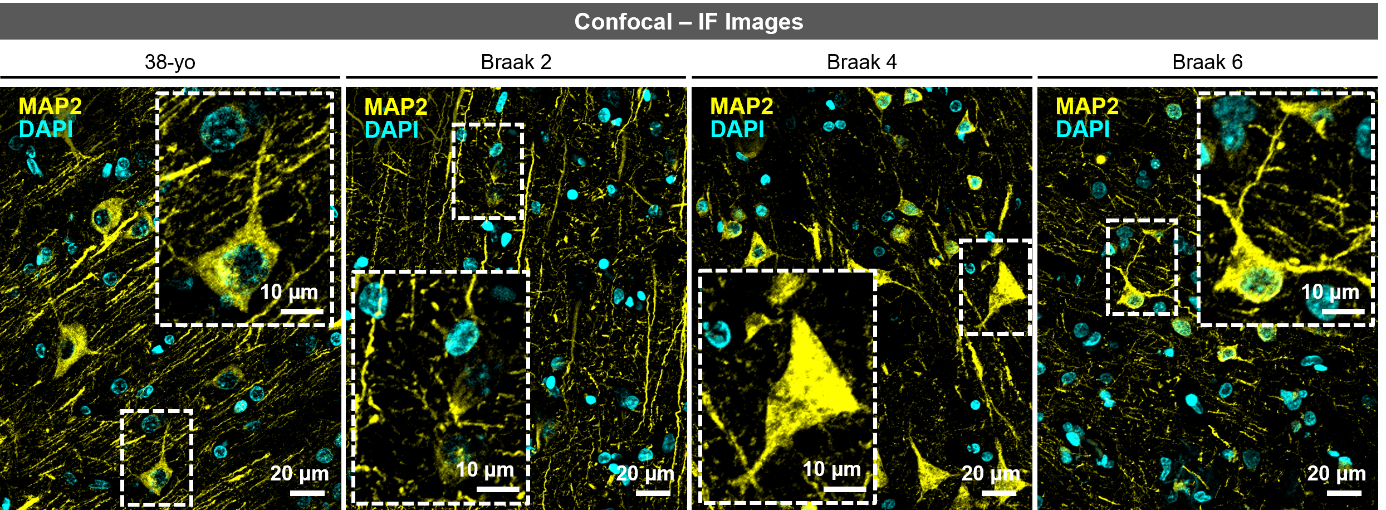


**Figure S2.** Neuronal cell bodies and processes were readily detectable across tissues. Representative single-color maximum z-projection confocal images of tissue sections from control (38-yo), PART (Braak 2), and AD (Braak 4 and 6) cases immunolabeled with MAP2 primary antibody. Yellow= MAP2, cyan= nuclei stained with DAPI. Images were acquired with 20x0.8NA objective and 0.3 µs pixel dwell time. 38-yo images were acquired using 405- and 488- nm lasers at (1.8% and 3.0%) laser power and (700 and 538) gain. Braak 2 images were acquired using 405- and 488- nm lasers at (1.8% and 1.2%) laser power and (700 and 508) gain. Braak 4 images were acquired using 405- and 488- nm lasers at (1.2% and 0.800%) laser power and (700 and 560) gain. Braak 6 images were acquired using 405- and 488- nm lasers at (1.8% and 3.0%) laser power and (700 and 549) gain. Brightness and contrast were adjusted individually to best visualize nuclei and cellular staining.


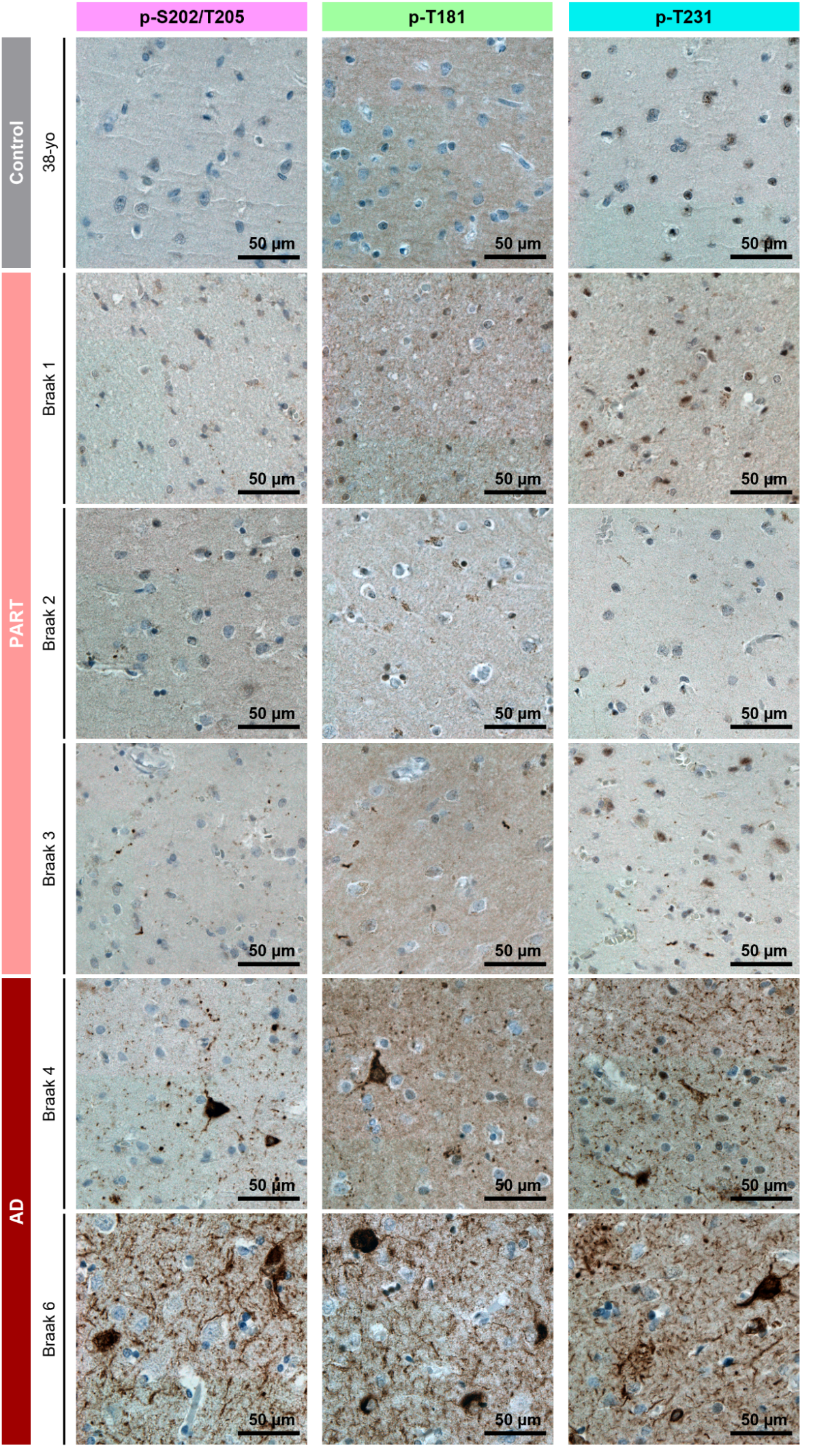


**Figure S3.** Immunohistochemistry (IHC) characterization of the cases. Representative widefield images of tissue sections from control (38-yo), PART (Braak 1-3), and AD (Braak 4 and 6) cases immunolabeled with AT8, AT270, and AT180 primary antibodies targeting hyperphosphorylated tau residues p-S202/T205, p-T181, and p-T231, respectively (brown) as well as DAPI staining of nuclei (blue). Acquisitions parameters) 40x0.95NA objective, LED-light source, gain-not applicable, 15 ms-exposure.


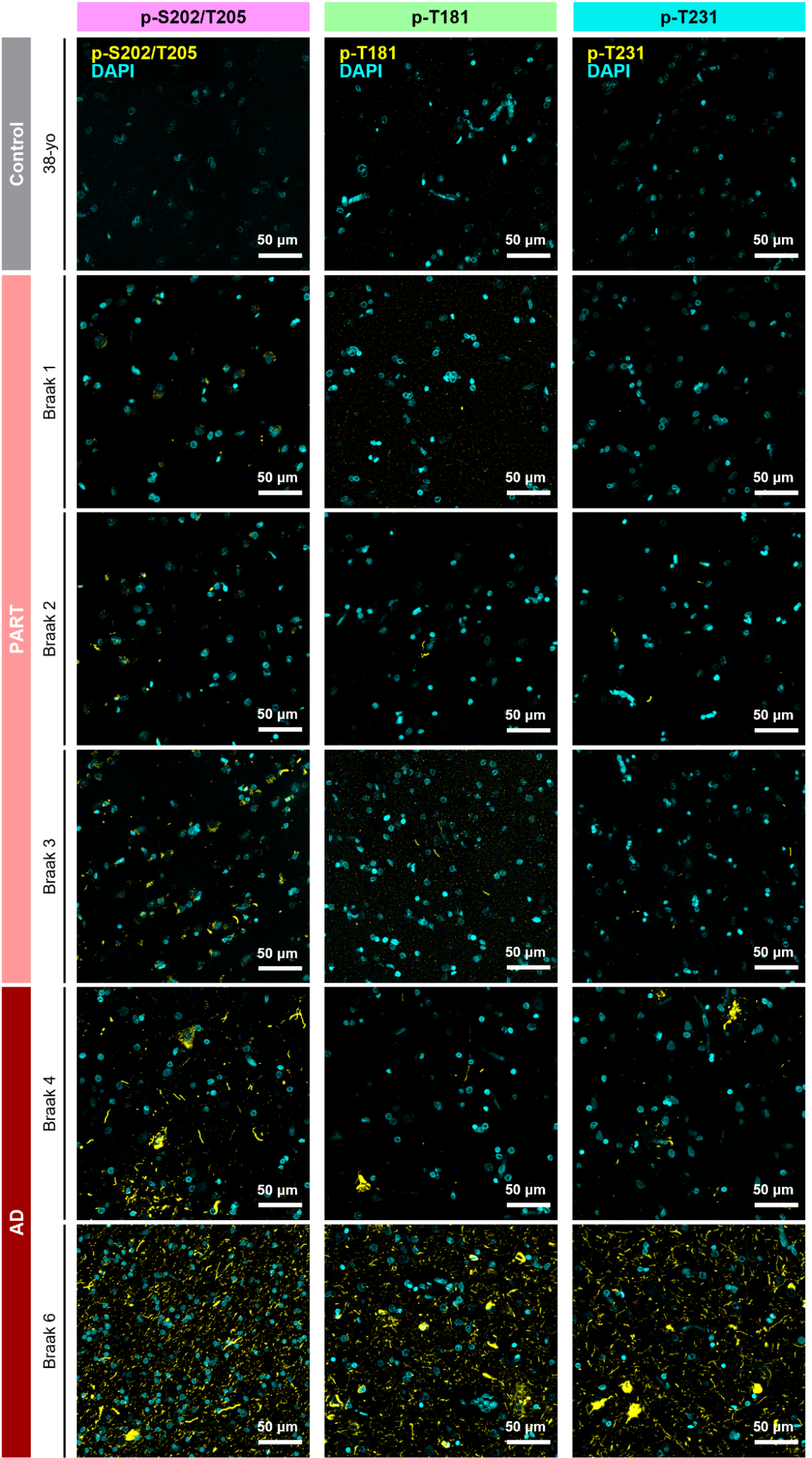


**Figure S4.** Immunofluorescence (IF) characterization of the cases. Representative single-color maximum z-projection confocal images of tissue sections from control (38-yo), PART (Braak 1-3), and AD (Braak 4 and 6) cases immunolabeled with AT8, AT270, and AT180 primary antibodies targeting hyperphosphorylated tau residues p-S202/T205, p-T181, and p-T231, respectively. Yellow= hyperphosphorylated tau (pS202/T205, p-T181, and p-T231), cyan= nuclei stained with DAPI. Images were acquired with 20x0.8NA objective and 0.3 µs pixel dwell time. p-S202/T205 IF images were acquired using 405- and 561- nm lasers at (0.500% and 1.8%) laser power and (700 and 730) gain, respectively. p-T181 IF images were acquired using 405- and 488-nm lasers at (0.700% and 0.900%) laser power and (700 and 500) gain, respectively. p-T231 IF images were acquired using 405- and 488-nm lasers at (0.600% and 0.600%) laser power and (690 and 510) gain, respectively.


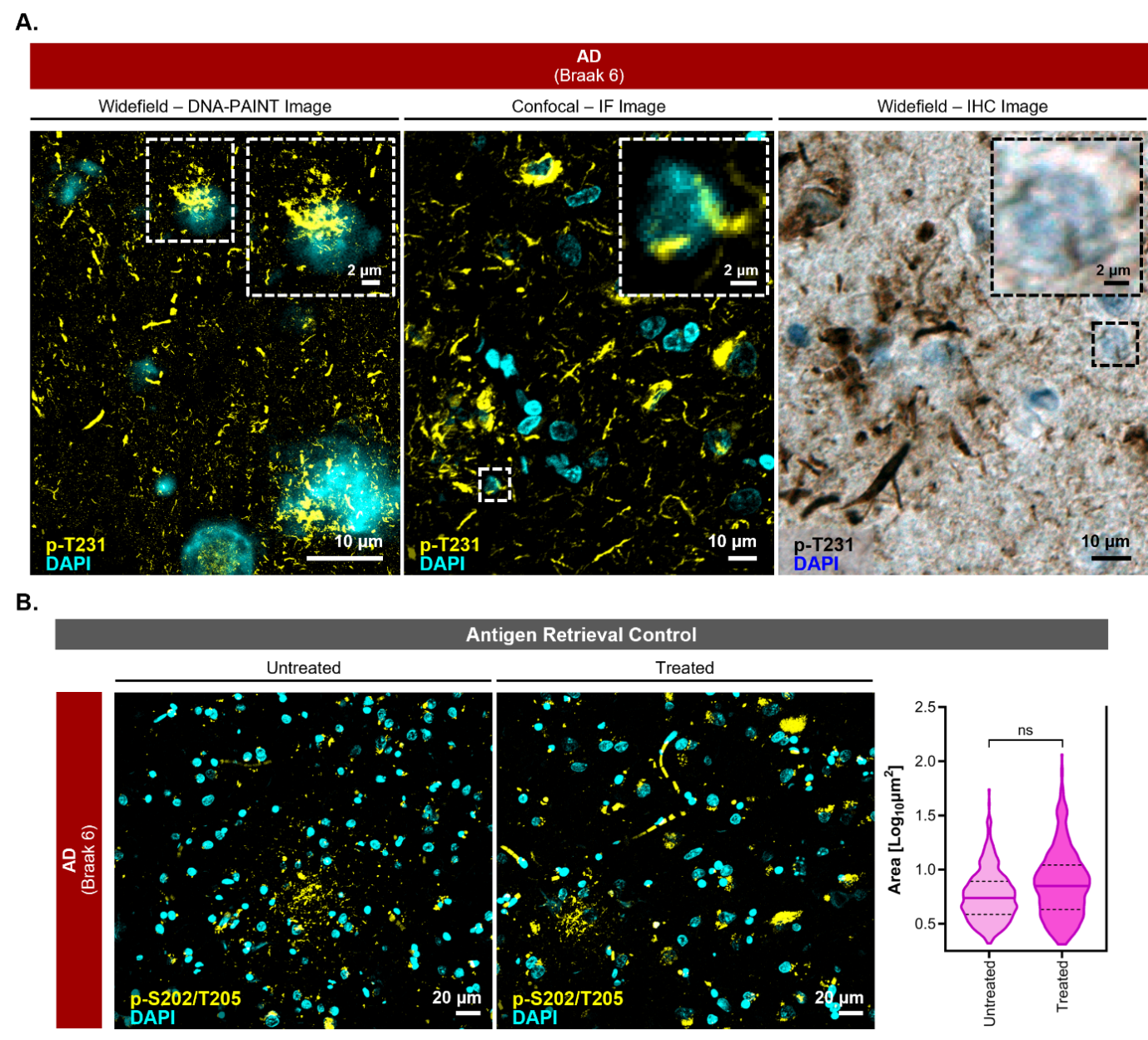


**Figure S5.** Staining patterns of p-tau aggregates are consistent across imaging modalities. Sample Size (B)) Untreated: N=1 tissue section, n=3 fields of view per section; Treated: N=1 tissue section, n=3 fields of view per section. Statistical Test (B)) An unpaired two-sided rank sum test was performed using the mean area value per field of view. A p value <0.05 was taken as statically significant. P values: ns (>0.05), * (0.05 – 0.01), ** (0.001 – 0.01), *** (0.0001 – 0.001), **** (< 0.0001). A) Left: Representative single-color DNA-PAINT image of p-T231 tau from an AD (Braak 6) tissue section immunolabeled with AT180 and stained with DAPI. Yellow = localizations of p-T231 tau and cyan = nucleus. Acquisition parameters: 100x/1.45NA oil immersion objective, 2.5 mW 561-nm lasers, gain-not applicable, 100-ms exposure time. Middle: Representative single-color maximum z-projection confocal IF image of p-T231 tau from an AD (Braak 6) tissue section immunolabeled with AT180 and stained with DAPI. Yellow = p-T231 tau and cyan = nucleus. Acquisition parameters: 20x0.8NA objective, 405- and 488-nm lasers, 690 gain and 0.6000% laser power (405-nm), 510 gain and 0.6000% laser power (488-nm), 0.3 µs pixel dwell time. Right: Representative widefield IHC image of p-T231 tau from an AD (Braak 6) tissue section immunolabeled with AT180 and stained with DAPI (blue). Insets highlight the presence of p-T231 tau accumulating around a nucleus, resembling the morphology of NFTs. Acquisition parameters: 40x0.95NA objective, LED-light source (10%), gain-not applicable, 15 ms-exposure time. B) AD (Braak 6) tissue sections were prepared including (treated) or omitting (untreated) the heat denaturation and antigen retrieval step, followed by immunolabeling with AT8 primary antibody and Alexa546 to image p-S202/T205 tau. Left: representative single-color maximum z-projection confocal IF image of p-S202/T205 tau (yellow) and nuclei (cyan) in untreated section. Right: representative single-color maximum z-projection confocal IF image of p-S202/T205 tau (yellow) and nuclei (cyan) in treated section. Confocal imaging parameters: 20x/0.8NA objective, 405- and 561-nm lasers, 700 gain and 0.5% laser power (405-nm), 750 gain and 2.8% laser power (561-nm), 0.3 µs pixel dwell time. Violin plots show the area distribution (log_10_μm) of the tau aggregates identified in images of AD (Braak 6) tissue sections immunolabeled with AT8. Solid line indicates median and dashed lines indicate the quartile percentiles (25th and 75^th^).


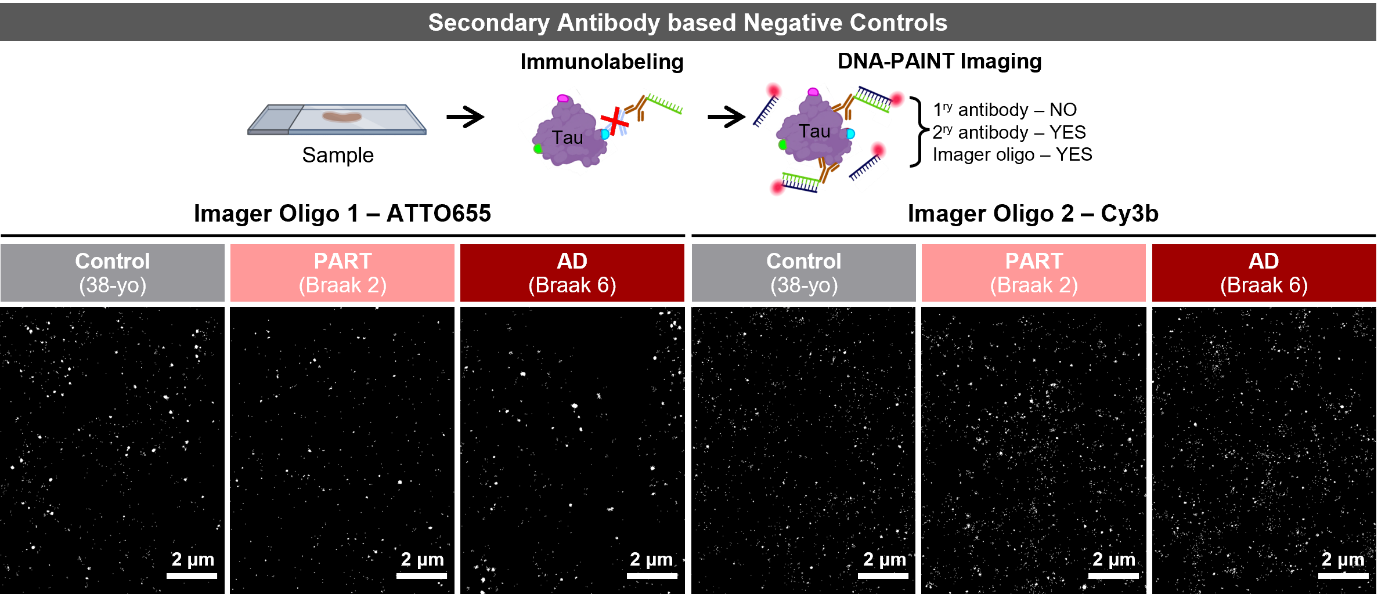


**Figure S6.** Single-color DNA-PAINT images of tau aggregates in human postmortem brain tissues. Representative single-color DNA-PAINT images of negative control samples. Sections from 38-yo control, PART (Braak 2), and AD (Braak 6) were immunolabeled and imaged using the pipeline described in Figure 1, but in the absence of phospho-specific primary antibodies. Representative images show sparse background localizations arising from non-specific labeling from secondary antibody and imager oligos. Mouse-specific secondary antibody (docking strand 1) was imaged with imager probe 1-ATTO655. Rabbit-specific secondary antibody (docking strand 2) was imaged with imager probe 2-Cy3b. Acquisition parameters: 100x/1.45NA oil immersion objective, 10 mW 647- and 2.5 mW 561-nm lasers, gain-not applicable, 100-ms exposure time.


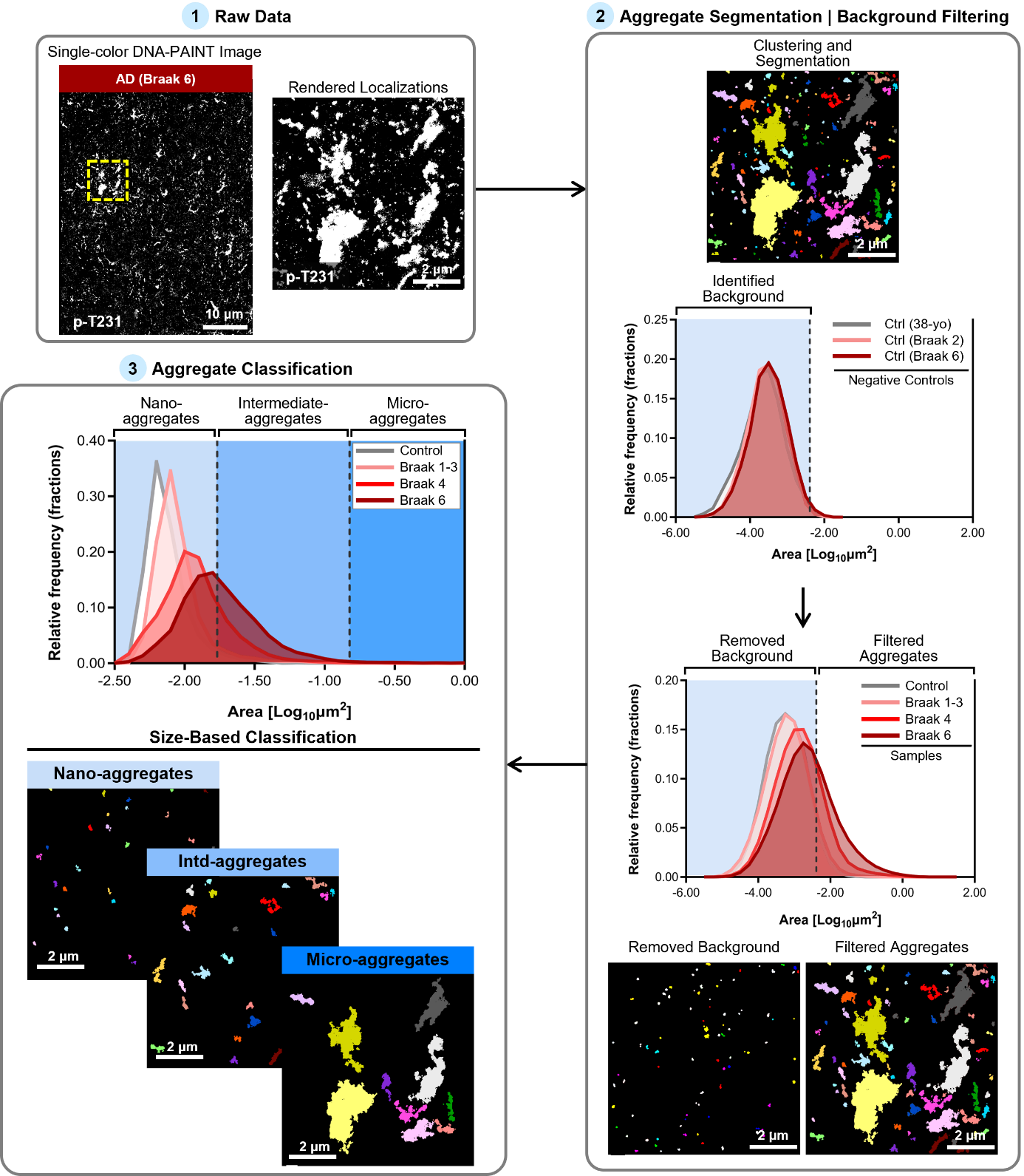


**Figure S7.** Pipeline for the identification and quantification of tau aggregates in single-color DNA-PAINT images. Sample Size) Control (38- and 60-yo) N=8 tissue sections (4 per case), n=32 collective fields of view; PART (Braak 1-3) N=16 tissue sections (4 per case), n=65 collective fields of view; AD (Braak 4) N=12 tissue sections (4 per case), n=49 collective fields of view; AD (Braak 6) N=11 tissue sections (4 for case A-B, 3 for case C), n=45 collective fields of view. Sample Size – Negative Controls) 38-yo, PART (Braak 2), AD (Braak 6), N=3 total tissue section (1 per case), n=4 fields of view per section. Representative single-color DNA-PAINT image of p-T231 in a section from AD (Braak 6) immunolabeled with AT180 antibody and imaged with imager probe 2-Cy3b. Acquisition parameters) 100x/1.45NA oil immersion objective, 2.5 mW 561-nm lasers, gain-not applicable, 100-ms exposure time. Inset shows a zoom-in of the yellow boxed region. 2) The point localizations are clustered and segmented using Voronoi Tessellation. Inset image shows corresponding Voronoi segmented clusters pseudo-color coded to identify individual p-T231 positive tau aggregates. Histogram shows the area (log_10_μm^2^) distribution of segmented clusters in images from negative control samples: 38-yo control (grey), PART (Braak 2) (light pink), and AD (Braak 6) (dark red). Light blue background highlights the size range of the identified clusters across negative controls. Dashed line indicates the area cutoff (-2.38 log_10_ μm^2^= 0.004 μm^2^) used for the area-based filtering step to remove background signal. Subsequent histogram shows the area distribution of segmented clusters in images from control (38-and 60-yo) (grey), PART (Braak 1-3) (light pink), AD (Braak 4) (dark pink), and AD (Braak 6) (dark red) samples. Light blue background highlights the region that falls below the area threshold established with the negative controls (dashed line). These clusters are considered background and removed from the list of segmented clusters. Insets show examples of the removed background signal and the filtered tau aggregates. 3) Histogram shows the area (log_10_μm^2^) distribution of filtered tau aggregates for p-T231 images from control (38-and 60-yo), PART (Braak 1-3), and AD (Braak 4 and 6) cases. All sections were immunolabeled with AT180 antibody and imaged with imager probe 2-Cy3b. Dashed lines accompanied with blue-colored shading boxes indicate the area-cut off used for size-based classification of tau aggregates: nano-aggregates (light blue; 0.004 – 0.017 μm^2^, corresponding to a radius of 35-60 nm assuming circular shape), intermediate-aggregates (periwinkle blue; 0.017 – 0.15 μm^2^) and micro-aggregates (dark blue; above 0.15 μm^2^). The upper limit of the x-axis was set to 0 for visual purposes. Insets show representative examples of tau aggregates belonging to each of the size-based classes, starting with nano-aggregates, intermediate-aggregates, and micro-aggregates.


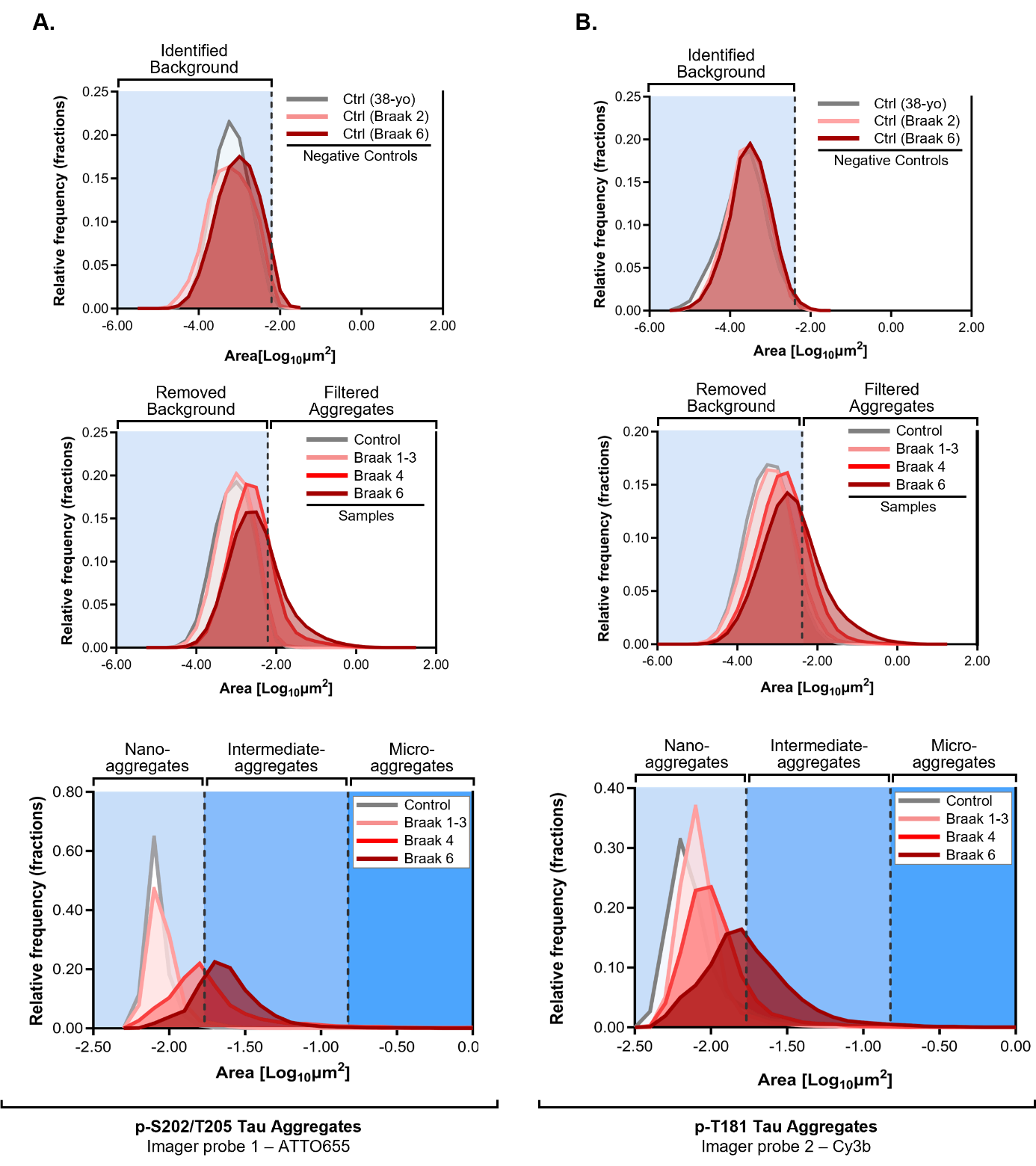


**Figure S8.** Negative control experiments for p-S202/T205 imaged with imager probe 1-ATTO655 and p-T181 imaged with imager probe 2-Cy3b. Sample Size – (A-B)) p-S202/T205, and pT181: p-S202/T205: Control (38-yo and 60-yo) N=16 tissue sections (8 per case), n=64 collective fields of view; PART (Braak 1-3) N=28 tissue sections (8 for Braak 1 case, 12 for Braak 2 cases, and 8 for Braak 3 case), n=112 collective fields of view; AD (Braak 4) N=20 tissue sections (4 for case A, 8 for cases B-C), n=79 collective fields of view; AD (Braak 6) N=18 tissue sections (4 for case A, 8 for case B, 6 for case C), n=78 collective fields of view. p-T181: Control (38- and 60-yo) N=8 tissue sections (4 per case), n=32 collective fields of view; PART (Braak 1-3) N=16 tissue sections from (4 per case), n=64 collective fields of view; AD (Braak 4) N=12 tissue sections (4 per case), n= 51 collective fields of view; AD (Braak 6) N=11 tissue sections (4 for cases A-B, 3 for case C), n=46 collective fields of view. Sample Size – (A-B)) Negative Controls p-S202/T205, and p-T181: 38-yo, PART (Braak2), AD (Braak 6) N=1 tissue section, n=4 fields of view per case.

A-B) Top: Histograms show the area (log_10_μm^2^) distribution of segmented clusters in images from negative control samples: 38-yo control (grey), PART (Braak 2) (light pink), and AD (Braak 6) (dark red), immunolabeled with (A) AT8 primary antibody or (B) AT270 antibody to identify p-S202/T205 and p-T181 tau, respectively. Light blue background highlights the size range of the identified clusters across negative controls. Dashed line indicates the area cutoff used for the area-based filtering step to remove background signal. Middle: Histograms (log_10_μm^2^) show the area distribution of segmented clusters in images of (A) p-S202/T205 and (B) p-T181 from control (38-and 60-yo) (grey), PART (Braak 1-3) (light pink), and AD (Braak 4, dark pink and Braak 6, dark red) samples. Light blue background highlights the region that falls below the area threshold established with the negative controls (dashed line). These clusters are considered background and removed from the list of segmented clusters. Bottom: Histograms show the area (log_10_μm^2^) distribution of filtered tau aggregates for (A) p-S202/T205 and (B) p-T181 images from control (38-and 60-yo) (grey), PART (Braak 1-3) (light pink), and AD (Braak 4, dark pink and 6, dark red) samples. The upper limit of x-axis was set to 0 for visual purposes. All sections immunolabeled with AT8 were imaged with imager probe 1-ATTO655 and all sections immunolabeled with AT270 were imaged with imager probe 2-Cy3b. Dashed lines accompanied with blue-colored shading boxes indicate the area-cut off used for size-based classification of tau aggregates into nano-, intermediate- and micro-aggregates. AT8 immunolabeled samples: nano-aggregates (light blue; 0.006 – 0.017 μm^2^), intermediate-aggregates (periwinkle blue; 0.017 – 0.15 μm^2^) and micro-aggregates (dark blue; above 0.15 μm^2^). AT270 immunolabeled samples: nano-aggregates (light blue; 0.004 – 0.017 μm^2^), intermediate-aggregates (periwinkle blue; 0.017 – 0.15 μm^2^) and micro-aggregates (dark blue; above 0.15 μm^2^).


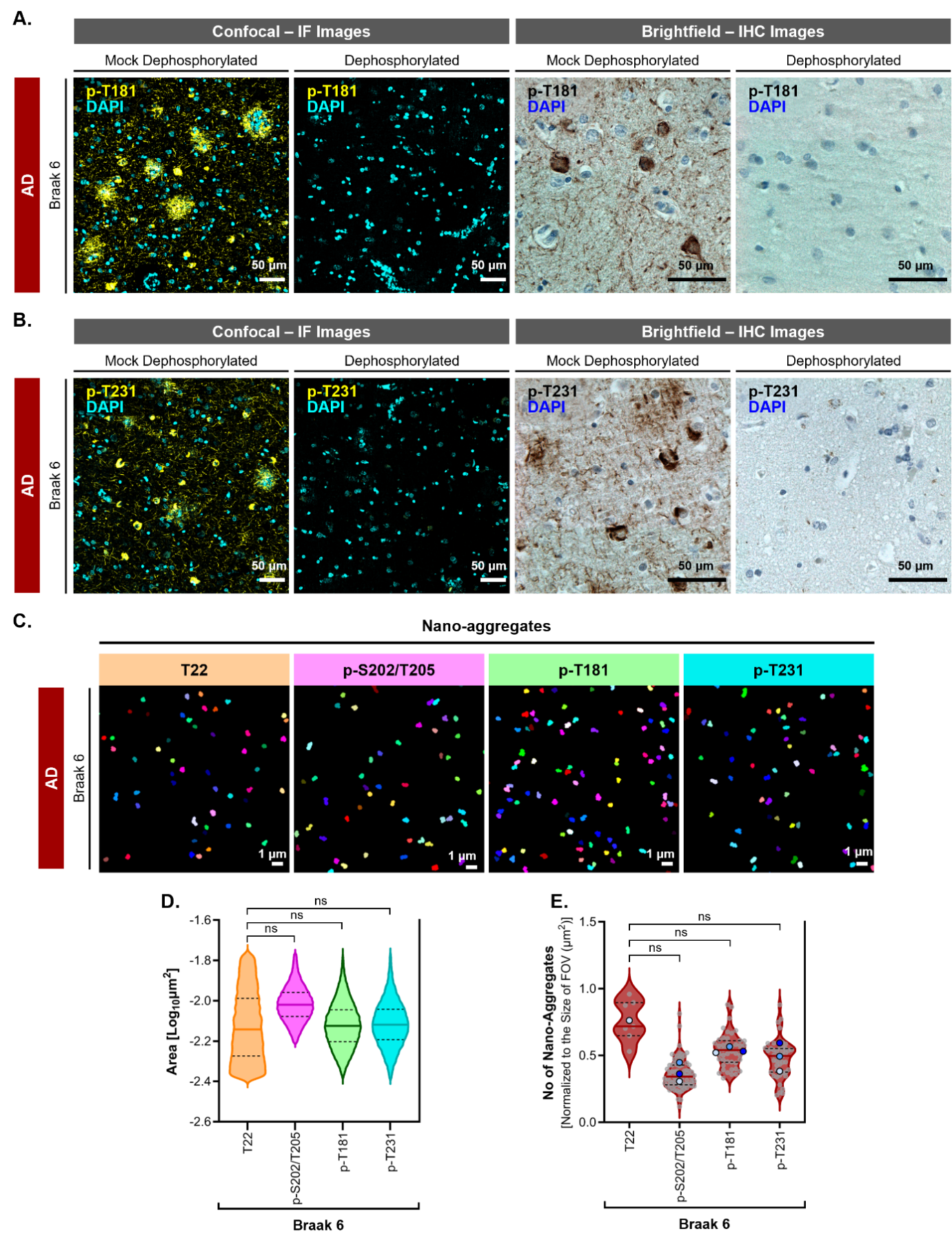


**Figure S9.** Validation experiments for the dephosphorylation assay and detection of nano-aggregates with oligomeric T22 antibody. Sample Size – (D-E)) T22, p-S202/T205, p-T181, and p-T231: T22: AD (Braak 6) N=1 tissue section from patient C, n=7 fields of view. p-S202/T205: AD (Braak 6) N=18 tissue sections (4 for case A, 8 for case B, 6 for case C), n=78 collective fields of view; p-T181: AD (Braak 6) N=11 tissue sections (4 for cases A-B, 3 for case C), n=46 collective fields of view. p-T231: AD (Braak 6) AD (Braak 6) N=11 tissue sections (4 for cases A-B, 3 for case C), n=45 collective fields of view. Statistical Tests – (D-E)) An unpaired two-sided rank sum test was performed using the means per case (blue-colored dots). A p value <0.05 was taken as statically significant. P values: ns (>0.05), * (0.05 – 0.01), ** (0.001 – 0.01), *** (0.0001 – 0.001), **** (< 0.0001). A) AD (Braak 6) tissue sections were mock dephosphorylated or treated with a dephosphorylating enzyme (dephosphorylated), followed by immunolabeling with AT270 primary antibody and Alexa488 or IHC antibodies to image p-T181 tau. Left: representative single-color maximum z-projection confocal IF images of p-T181 tau (yellow) and nuclei (cyan) in mock treated and enzyme treated sections; Right: representative widefield IHC images (40x0.95NA) of p-T181 tau in mock treated and enzyme treated sections. Both IF and IHC confirm a drastic decrease in p-T181 signal in enzyme treated sections. Confocal imaging parameters: 20x/0.8NA objective, 405- and 488-nm lasers, 600 gain and 4% laser power (405-nm), 545 gain and 0.600% laser power (488-nm), 0.3 µs pixel dwell time. Widefield acquisition parameters: parameters: 40x/0.95NA objective, LED-light source (10%), gain-not applicable, 15 ms-exposure. B) AD (Braak 6) tissue sections were mock treated or treated with a dephosphorylating enzyme, followed by immunolabeling with AT180 primary antibody and Alexa488 or IHC antibodies to image p-T231 tau. Left: representative single-color maximum z-projection confocal IF images (20x0.8NA) of p-T231 tau (yellow) and nuclei (cyan) in mock treated and enzyme treated sections; Right: representative widefield IHC images (40x0.95NA) of p-T231 tau in mock treated and enzyme treated sections. Both IF and IHC confirm drastic decrease in p-T231 signal in enzyme treated sections. Confocal imaging parameters: 20x/0.8 NA objective, 405- and 488-nm lasers, 600 gain and 5% laser power (405-nm), 545 gain and 0.700% laser power (488-nm), 0.3 µs pixel dwell time. Widefield acquisition parameters: 40x/0.95NA objective, LED-light source (10%), gain-not applicable, 15 ms-exposure. C) Representative images of pseudo-colored segmented tau nano-aggregates from AD (Braak 6) tissue samples immunolabeled with primary antibodies T22, AT8, AT270, and AT180. D) Violin plots show the area distribution (log10 μm^2^) of the tau nano-aggregates segmented from images of AD (Braak 6) tissue sections immunolabeled with T22, AT8, AT270, and AT180. The area of nano-aggregates detected by each antibody is within a similar range. E) Violin plots show the distribution of the mean number of tau nano-aggregates segmented from images of AD (Braak6). The number of nano-aggregates detected per unit area with each antibody is within a similar range. D-E) Solid line indicates median and dashed lines indicate the quartile percentiles ( 25th and 75^th^). Small grey dots represent the mean number of aggregates per field of view and larger dots represent the mean number of aggregates per patient, starting with patient A (light blue), patient B (periwinkle blue), patient C (dark blue).


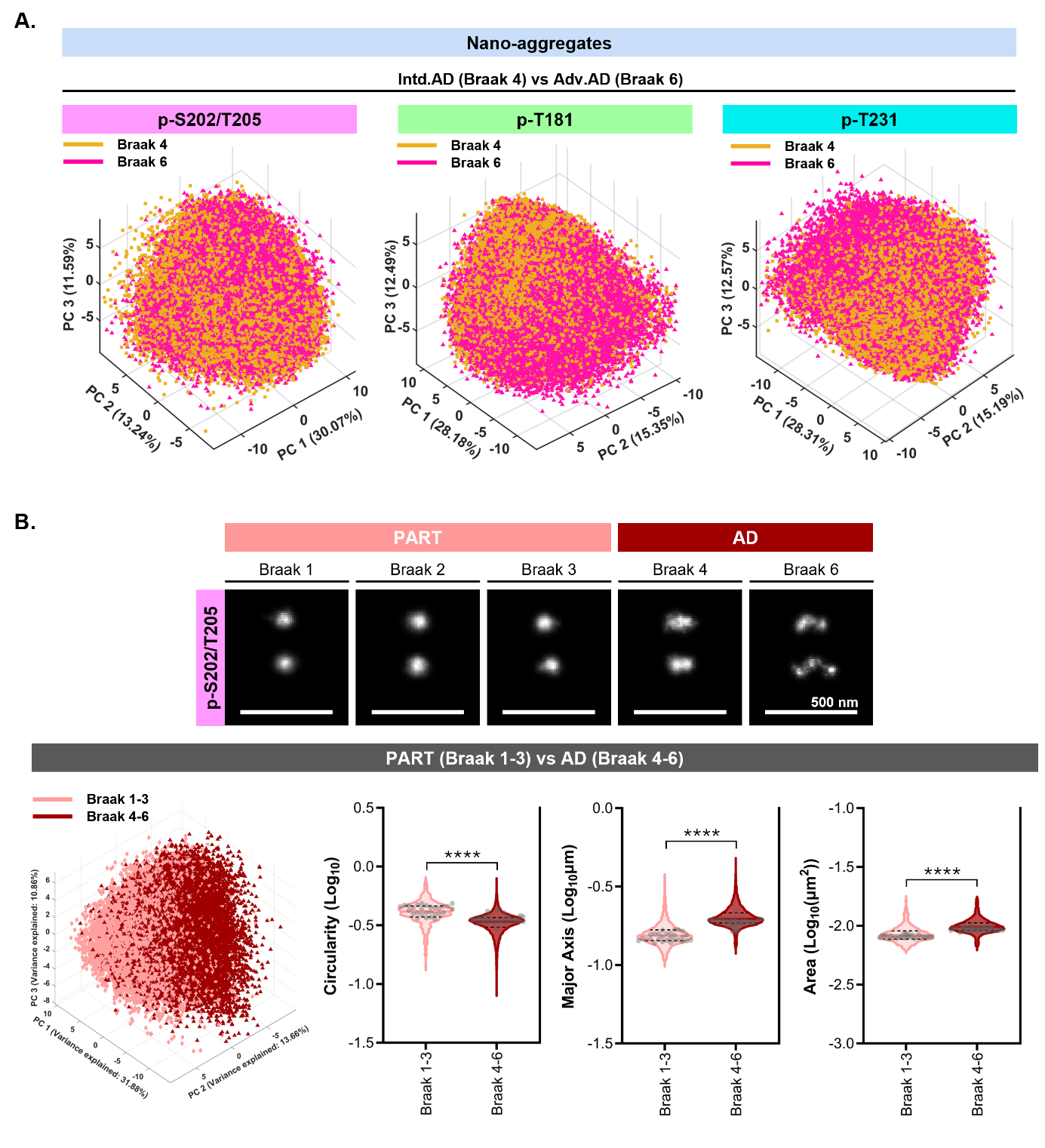


**Figure S10.** Morphology analysis comparing tau nano-aggregates between Intermediate and Advanced AD and p-T202/S205 labeled tau nano-aggregates between PART and AD. Sample Size – (A)) p-S202/T205, p-T181, and p-T231: p-S202/T205: AD (Braak 4) N=20 tissue sections (4 for case A, 8 for cases B-C), n=79 collective fields of view; AD (Braak 6) N=18 tissue sections (4 for case A, 8 for case B, 6 for case C), n=78 collective fields of view. p-T181: AD (Braak 4) N=12 tissue sections (4 per case), n=51 collective fields of view; AD (Braak 6) N=11 tissue sections (4 for cases A-B, 3 for case C), n=46 collective fields of view. p-T231: AD (Braak 4) N=12 tissue sections (4 per case), n=49 collective fields of view; AD (Braak 6) N=11 tissue sections (4 for cases A-B, 3 for case C), n=45 collective fields of view. Sample Size – (B)) p-S202/T205: PART (Braak 1-3) N=28 tissue sections from 4 cases, n=112 fields of view; AD (Braak 4-6) N=38 tissue sections from 6 cases, n=157 collective fields of view. Statistical Tests – (B)) An unpaired two-sided rank sum test was performed using the means per field of view (light grey dots). A p value <0.05 was taken as statically significant. P values: ns (>0.05), * (0.05 – 0.01), ** (0.001 – 0.01), *** (0.0001 – 0.001), **** (< 0.0001). A) Principal Component Analysis (PCA) plots showing the first three principal components for p-S202/T205, p-T181, and p-T231 nano-aggregates in Intermediate AD (Braak 4) and Advanced AD (Braak 6) cases (yellow = Braak 4 and magenta = Braak 6). B) Representative examples of p-S202/T205 immunolabeled nano-aggregates found in images from PART (Braak 1-3) and AD (Braak 4 and 6) samples. Principal Component Analysis (PCA) plots show the first three principal components for p-S202/T205 immunolabeled nano-aggregates in PART (light pink; Braak 1-3) and AD (dark pink; Braak 4 and 6). Violin plots show the distribution of p-S202/T205 immunolabeled nano-aggregates’ circularity (log_10_), major axis (log_10_μm), and area (log_10_μm^2^) in PART (light pink; Braak 1-3), and AD (dark pink; Braak 4 and 6) samples. Solid line indicates median, and the dashed lines indicate the quartile percentiles ( 25th and 75th). Small grey dots represent the mean value (circularity, major axis, and area) per replicate (tissue section).


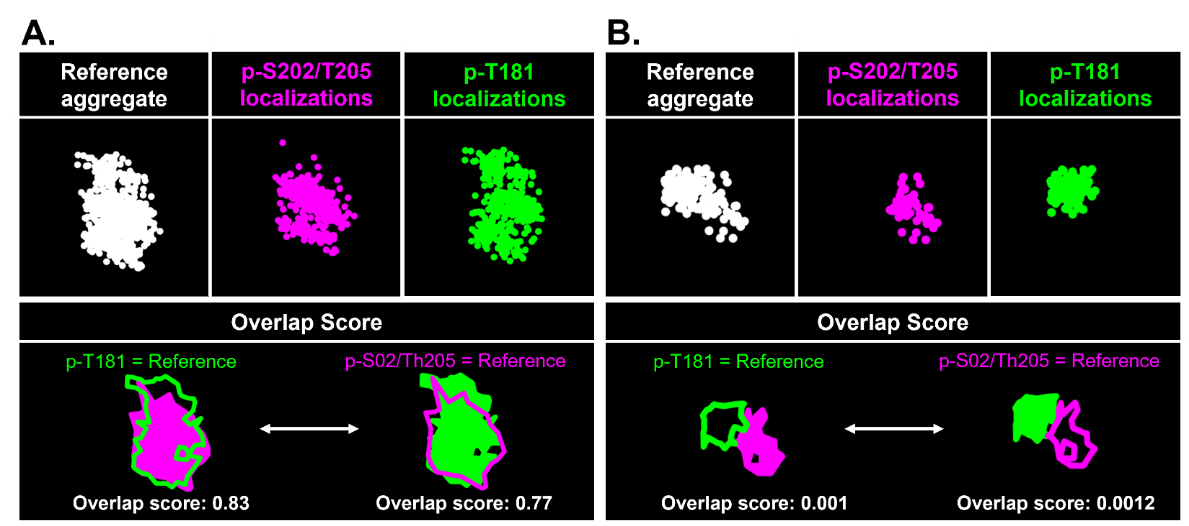


**Figure S11.** Quality control step for segmentation in dual-color DNA-PAINT images. Occasionally, when two distinct tau aggregates appeared in separate channels but were spatially proximate, they might overlap partially in the merged reference image and thus be segmented as a single tau aggregate. To rectify such segmentation errors, we assessed the percentage of overlap of each individual channel within the segmented reference tau aggregate. A high percentage overlap suggests that the detected localizations from each channel intermingle and coincide within the segmented reference aggregate, likely indicating the presence of a single aggregate with a mixed modification profile as shown in panel A. Conversely, a low percentage overlap indicates that the detected localizations from each channel remain separate within the segmented reference aggregate, suggesting two distinct aggregates that contain a single modification and that partially overlap in space as shown in panel B. Therefore, we conducted this quality control step to ascertain the overlap score of segmented tau aggregates in dual-color DNA-PAINT images from PART and AD. If the overlap score fell below 30%, segmented reference aggregate is separated and the localizations from each channel are re-segmented separately. Following this quality control step, tau aggregates exhibited an average high overlap score of 67%, confirming the effectiveness of the segmentation. Representative example of a properly segmented tau nano-aggregate with high overlap score is shown in (A) and a segmentation error with low overlap score is shown in (B). White shows the reference tau aggregate localized points after segmentation from the merged reference image. Magenta shows the localized points corresponding to the p-S202/T205 channel. Green shows the localized points corresponding to the p-T181 channel. Magenta and green outlines show the alpha-shape computed from each channel overlaid on top of the other channel. A high overlap of the two alpha-shapes gives rise to a high overlap score and vice versa.


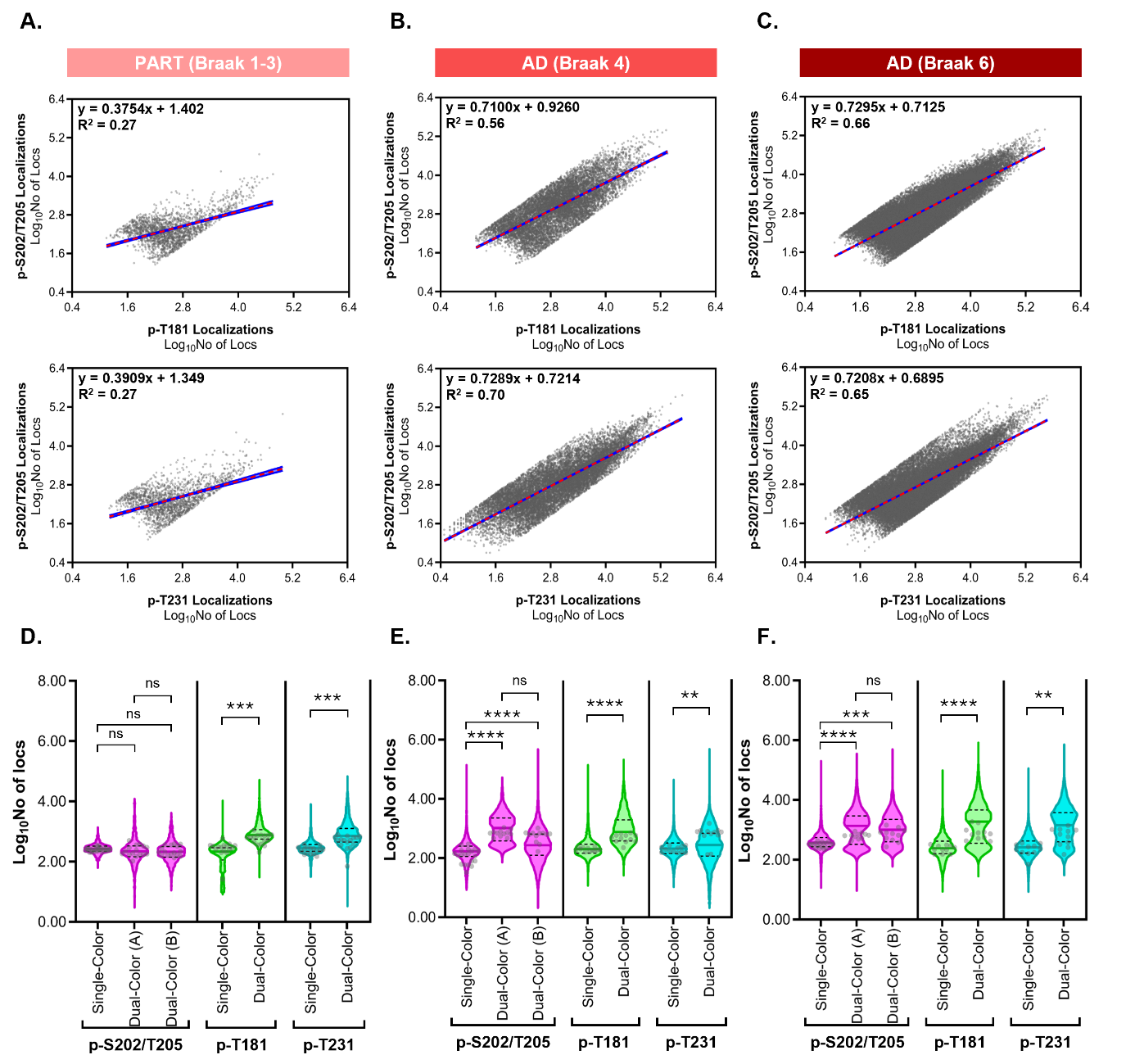


**Figure S12.** DNA-PAINT dual-color imaging controls. Sample Size – (A-C)) p-S202/T205 + p-T181, and p-S202/T205 + p-T231: p-S202/T205 + p-T181: PART (Braak 1-3) N=16 sections (4 per case), n=63 collective fields of view; AD (Braak 4) N=12 tissue sections (4 per case), n=51 collective fields of view; AD (Braak 6) N=11 tissue sections (4 for case A-B, 3 for case C), n=43 collective fields of view. p-S202/T205 + p-T231: PART (Braak 1-3), N=16 sections (4 per case), n=65 collective fields of view; AD (Braak 4) N=12 tissue sections (4 per case), n=50 collective fields of view; AD (Braak 6) N=11 tissue sections (4 for case A-B, 3 for case C), n=44 collective fields of view. Sample Size – (D-F)) p-S202/T205, p-T181, and p-T231 single-color samples: p-S202/T205 Samples: PART (Braak 1-3) N=28 tissue sections (8 for Braak 1 case, 12 for Braak 2 cases, and 8 for Braak 3 case), n=112 collective fields of view; AD (Braak 4) N=20 tissue sections (4 for case A, 8 for cases B-C), n=79 collective fields of view; AD (Braak 6) N=18 tissue sections (4 for case A, 8 for case B, 6 for case C), n=78 collective fields of view. p-T181 Samples: PART (Braak 1-3) N=16 tissue sections from (4 per case), n=64 collective fields of view; AD (Braak 4) N=12 tissue sections (4 per case), n= 51 collective fields of view; AD (Braak 6) N=11 tissue sections (4 for cases A-B, 3 for case C), n=46 collective fields of view. p-T231 Samples: PART (Braak 1-3) N=16 tissue sections (4 per case), n=65 collective fields of view; AD (Braak 4) N=12 tissue sections (4 per case), n=49 collective fields of view; AD (Braak 6) N=11 tissue sections (4 for cases A-B, 3 for case C), n=45 collective fields of view. p-S202/T205, p-T181, and p-T231 from dual-color samples: p-S202/T205 and p-T181: PART (Braak 1-3) N=16 sections (4 per case), n=63 collective fields of view; AD (Braak 4) N=12 tissue sections (4 per case), n=51 collective fields of view; AD (Braak 6) N=11 tissue sections (4 for case A-B, 3 for case C), n=43 collective fields of view. p-S202/T205 and p-T231 Samples: PART (Braak 1-3), N=16 sections (4 per case), n=65 collective fields of view; AD (Braak 4) N=12 tissue sections (4 per case), n=50 collective fields of view; AD (Braak 6) N=11 tissue sections (4 for case A-B, 3 for case C), n=44 collective fields of view. Statistical Tests – (D-F)) An unpaired two-sided rank sum test was performed using the means per replicate (light grey dots). A p value <0.05 was taken as statically significant. P values: ns (>0.05), * (0.05 – 0.01), ** (0.001 – 0.01), *** (0.0001 – 0.001), **** (< 0.0001). It is possible that steric hindrance effects could preclude the simultaneous binding of two antibodies to adjacent modified residues. To confirm this did not occur, we assessed the number of detected localizations within tau aggregates for each modification in dual-color images from all samples (PART and AD). If steric hindrance posed a problem, we would anticipate a negative correlation between the number of detected localizations (i.e., high detection for one modification correlating with low detection for the other). However, in most cases we observed a positive correlation between the localizations detected for combinations of phospho-tau modifications as shown in panels A and B. A-C) X,Y scatter plot showing the number of localizations of p-T181 and p-S202/T205 or p-T231 and p-S202/T205 per tau aggregate in images from PART (Braak 1-3), AD (Braak 4), and AD (Braak 6) samples. The red line is the linear regression, and the blue shaded area is the standard error. D-F) Violin plots show the number of localizations per tau aggregate for p-S202/T205 (magenta), p-T181 (green), and p-T231 (cyan) from single-color or dual-color DNA-PAINT images in (D) PART (Braak 1-3), (E) AD (Braak 4), (F) and AD (Braak 6). Solid line indicates median and dashed lines indicate the quartile percentiles (25th and 75th). Small grey dots represent the mean number of localizations of p-tau aggregates per replicate (tissue section). p-S202/T205 has two plots for dual-color images corresponding to the two combinations, one for p-S202/T205 with p-T181 (Dual-Color A), and the second for pS202/T205 with p-T231 (Dual-Color B), respectively.
